# Stem cell-derived extracellular vesicles inhibit and revert fibrosis progression in a mouse model of diabetic nephropathy

**DOI:** 10.1038/s41598-019-41100-9

**Published:** 2019-03-14

**Authors:** Cristina Grange, Stefania Tritta, Marta Tapparo, Massimo Cedrino, Ciro Tetta, Giovanni Camussi, Maria Felice Brizzi

**Affiliations:** 1 Department of Medical Sciences, University of Turin, Turin, Italy; 2 Molecular Biotechnology Centre, University of Turin, Turin, Italy; 3 Unicyte Srl, Turin, Italy; 4 2i3T Società per la gestione dell’incubatore di imprese e per il trasferimento tecnologico Scarl, University of Turin, Turin, Italy

## Abstract

Extracellular vesicles (EVs) that are derived from mesenchymal stromal cells (MSCs) have been shown to reprogram injured cells by activating regenerative processes. We herein investigate the potential therapeutic effect of EVs, shed by human bone marrow MSCs and by human liver stem-like cells (HLSCs), on the progression and reversion of fibrosis in a mouse model of diabetic nephropathy, as induced by streptozotocin. After the development of nephropathy, stem cell-derived EVs were administered weekly to diabetic mice for four weeks. The stem cell-derived EV treatment, but not the fibroblast EV treatment that was used as a control, significantly ameliorated functional parameters, such as albumin/creatinine excretion, plasma creatinine and blood urea nitrogen, which are altered in diabetic mice. Moreover, the renal fibrosis that develops during diabetic nephropathy progression was significantly inhibited in stem cell EV-treated animals. A correlation was found between the down regulation of several pro-fibrotic genes in renal tissues and the anti-fibrotic effect of HLSC and MSC EVs. A comparative analysis of HLSC and MSC EV miRNA content highlighted some common and some specific patterns of miRNAs that target predicted pro-fibrotic genes. In conclusion, stem cell-derived EVs inhibit fibrosis and prevent its progression in a model of diabetes-induced chronic kidney injury.

## Introduction

Diabetic nephropathy (DN), a long-term diabetes complication, is the most common cause of end-stage chronic kidney disease (CKD) and has a high mortality rate^1^. The incidence of diabetes is dramatically increasing and is expected to rise to over 640 million patients by 2040 in urbanized countries^2^. Notably, DN affects about one-third of diabetic patients and this microvascular complication is a major health problem worldwide^3^. Hemodialysis and transplantation are the current therapeutic treatments for end-stage CKD, however both approaches have limitations, including high costs and organ availability^4^.

Hyperglycemia induces oxidative stress, with the overproduction of reactive oxygen species, as well as inflammation, with the release of pro-inflammatory cytokines and the recruitment of inflammatory cells. This results in glomerular and tubule-interstitial fibrosis, which is one of the hallmarks of DN and also considered to be the leading cause of renal dysfunction^5,6^. The progression of the fibrotic process correlates with the decline in renal function^7^. The epithelial-to-mesenchymal transition (EMT) is a crucial process that occurs during fibrosis, even in kidney, and stimulates the activation of myofibroblasts. This translates into the *de novo* deposition of extracellular matrix molecules, such as collagen I, fibronectin and laminin^8,9^. The transforming growth factor β (TGF-β) superfamily plays a significant role in modulating fibrosis^10^.

In this scenario, novel therapeutic strategies that can prevent the decline of renal function and reduce the progression of fibrosis are therefore in high demand. Of the new innovative approaches available, extracellular vesicles (EVs) appear to show particular promise. EVs that are shed by mesenchymal stromal cells (MSCs) have been described to modulate proliferation, angiogenesis and immune escape, which are all involved in tissue regeneration^11–13^. EVs are a heterogeneous population of circular membrane fragments that are released by most cell types as exosomes and/or shedding vesicles. EVs can be instrumental in cell-to-cell communication as they transfer their cargo of several RNA types (mRNAs, miRNAs, long-non coding-RNAs, tRNAs, rRNAs, circular-RNAs and piRNAs), proteins and bioactive lipids from the origin cell to target cell. Stem cell-derived EVs may also induce epigenetic changes in injured recipient cells with the activation of regenerative programs^14,15^. Indeed, it has been shown that EVs, released by MSCs of varying origin (bone marrow, liver, adipose tissue, renal, cord blood and Warthon’s jelly), accelerate renal repair in models of acute kidney injury (AKI)^16–22^. Moreover, multiple injections of MSC EVs were also shown to reduce the progression of renal injury in experimental models of CKD that was induced using five-sixth resection and unilateral ureteral obstruction^23,24^. Kholia *et al*.^25^ have recently shown that EVs, derived from human liver stem-like cells (HLSCs), significantly inhibit reductions in renal function and prevent fibrosis in a model of aristolochic-acid-induced CKD. Previous studies have demonstrated that HLSCs, a multipotent population of adult stem cells derived from human livers that express MSC and embryonic markers, release EVs with pro-regenerative activity, not only in the liver, but also in the kidney^19,26,27^. Moreover, EVs derived from stem cells purified from human urine, have been described as effective in the prevention of kidney injury by inhibiting apoptosis when administered before the establishment of DN, in a rat model of diabetes generated via streptozotocin (STZ) injection^28^. The conditioned medium that derives from MSCs has also been shown to improve DN via the presence of paracrine factors that included exosomes; in fact, a single administration of exosomes drove a rapid improvement in renal morphology^8^. However, there is currently no data on the reversal of DN via treatment with stem cell-derived EVs.

The aim of this study is therefore to investigate whether EVs, released by human bone marrow MSCs and HLSCs, may be beneficial in preventing and reverting DN progression in NOD/SCID/IL2Rγ KO (NSG) mice.

## Results

### *In vivo* model of diabetic nephropathy induced by STZ

**Figure 1:**
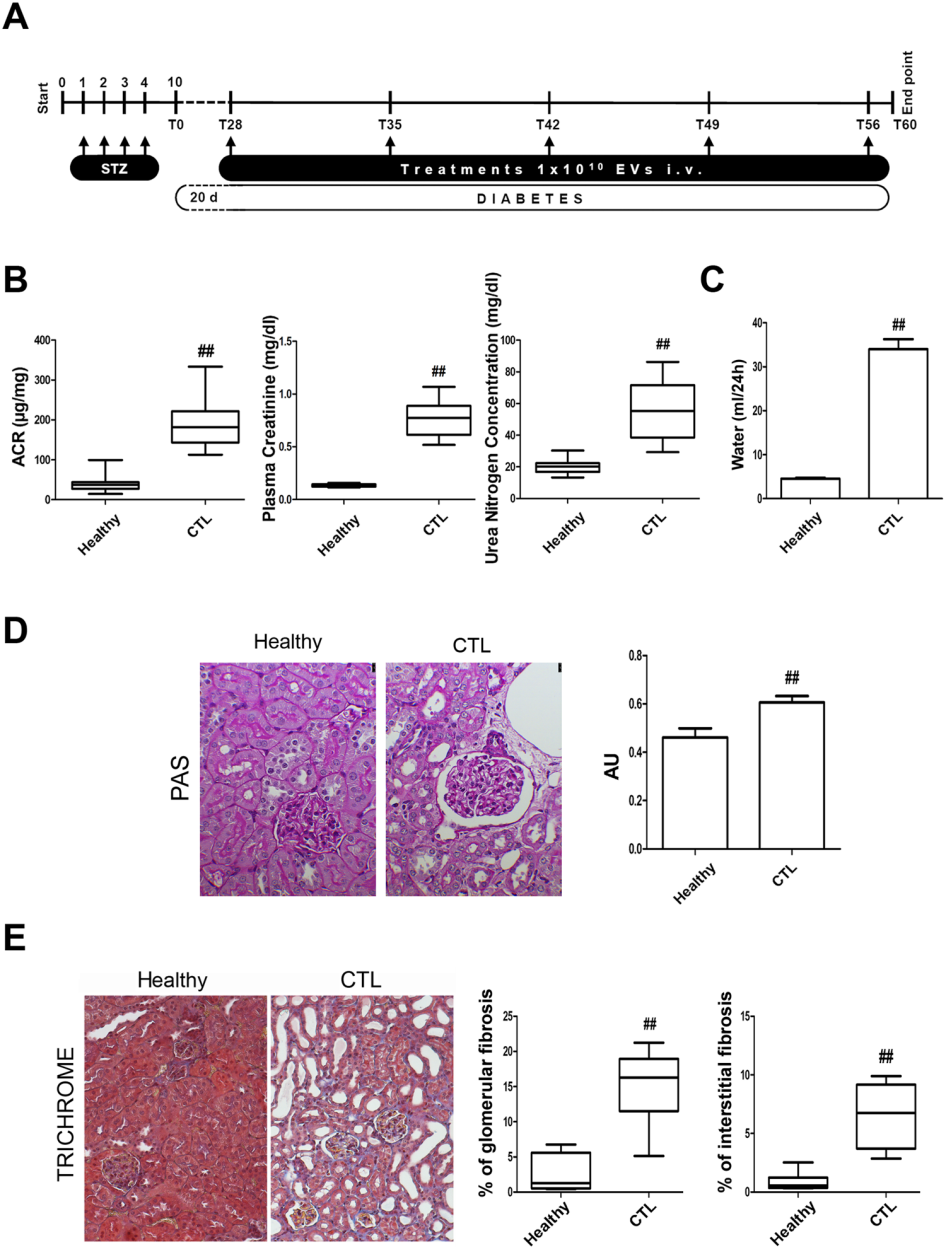
Induction of diabetic nephropathy in NSG mice by STZ injection. (**A**) Schematic representation of the experimental protocol for the establishment of DN and treatment with EVs. (**B**) ACR, plasma creatinine and BUN in healthy and STZ-diabetic mice (CTL) analyzed 28 days (T28) after the onset of diabetes. Data are expressed as mean ± SEM; ^##^p < 0.001 versus healthy (n = 10). (**C**) Histogram of the water uptake per mouse per 24 hours expressed as mean ± SEM; ^##^p < 0.001 versus healthy (n = 10). Student’s t-test was performed. (**D**) Representative images of renal PAS stained sections of healthy and STZ-diabetic mice (CTL) (magnification: 400X) and quantification of Bowman’s space. Data are expressed in arbitrary units as mean ± SEM; ^##^p < 0.001 versus healthy (n = 10). Student’s t-test was performed. (**E**) Representative images of renal Masson’s trichrome sections of healthy mice and STZ-diabetic mice (CTL) and quantification of glomerular and interstitial fibrosis (magnification: 200X). Data are expressed as percentage of fibrosis/glomerular area and as percentage of fibrosis/total area, respectively. Data are representative of mean ± SEM; ^##^p < 0.001 versus healthy (n = 10). Student’s t-test was performed.

DN was generated in male NSG mice via the intraperitoneal injection of 37 mg/kg of STZ for 4 consecutive days. The mice developed hyperglycemia (355 ± 85 mg/dl of glucose) ten days after STZ injection (T0, Fig. 1A). Glycaemia was monitored every 2 weeks and glucose levels were constantly high in all mice throughout the experimental period (≥350 mg/dl of glucose, not shown). On day 28 after the onset of diabetes, urine albumin excretion, estimated as the albumin-to-creatinine ratio (ACR), was significantly higher in STZ-diabetic mice (CTL) than in healthy mice (Fig. 1B). Only healthy mice displayed constant weight gain, whereas the body weight of STZ-diabetic mice decreased significantly (not shown). Plasma creatinine and blood urea nitrogen (BUN) levels were found to be significantly higher in STZ-diabetic mice than in healthy ones, thus confirming renal damage (Fig. 1B). Moreover, water uptake was elevated in STZ-diabetic mice (Fig. 1C), which is in line with persistent hyperglycemia. Periodic Acid-Schiff (PAS) staining showed an increase in Bowman’s space in STZ-diabetic mice (Fig. 1D) in accordance with DN-associated renal histopathological changes. Moreover, trichrome staining revealed alterations in renal collagen deposition in the glomerular and interstitial renal spaces of STZ-diabetic mice (Fig. 1E).

### EVs released by HLSCs and MSCs, but not by fibroblasts, ameliorate renal dysfunction

**Figure 2:**
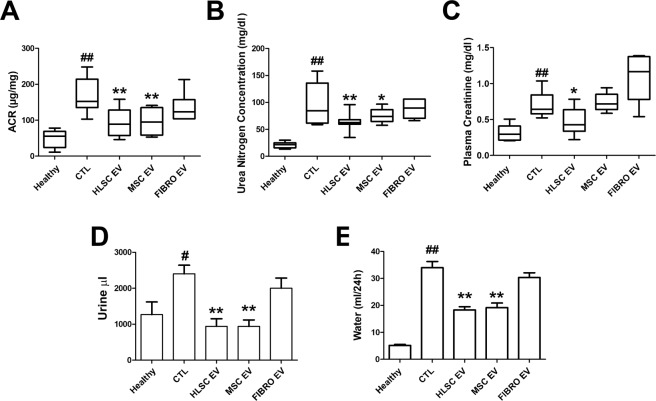
HLSC and MSC EVs improved renal function in STZ-induced diabetic nephropathy. ACR (**A**), BUN (**B**) and plasma creatinine (**C**) in healthy, CTL and EV-treated (HLSC EV, MSC EV and FIBRO EV) STZ-diabetic mice, analyzed 60 days (T60) after the onset of diabetes. Data are expressed as mean ± SEM; ^##^p < 0.001 versus healthy; *p < 0.05 and **p < 0.001 versus CTL (n = 8). ANOVA with Dunnet’s multi comparison test was performed. Histograms of urine excretion (μl/24 h) (**D**) and water uptake (ml/24 h) (**E**) per mouse, expressed as mean ± SEM; ^#^p < 0.05 and ^##^p < 0.001 versus healthy; *p < 0.05 and **p < 0.001 versus CTL (n = 8). ANOVA with Dunnet’s multi comparison test was performed. No significant differences were observed with CTL and FIBRO EV.

We evaluated whether the EVs released from HLSCs and MSCs may revert functional and histopathological alterations caused by DN in STZ-induced diabetic mice. The experimental protocol is shown in Fig. 1A. EVs derived from HLSCs (HLSC EVs), MSCs (MSC EVs) and from fibroblasts (FIBRO EVs), the last of which were used as a control, were injected intravenously from day 28 (T28), once a week for 4 consecutive weeks (5 injections) at a dose of 1 × 10^10^ EVs/each injection. The dose and timing were set up according to preliminary experiments that were performed to define the number of EVs that was required to obtain the best biological response (not shown). EV treatment started when the ACR was elevated and the histopathological signs of DN were already evident. EV treatment did not ameliorate either hyperglycemia or body weight (not shown). Mice were sacrificed 4 days after the last administration of EVs (T60, from the onset of diabetes). Stem cell-derived EV treatment considerably improved renal function (Fig. 2A) despite being ineffective in reducing plasma glucose concentrations. Both HLSC and MSC EVs induced a significant reduction in ACR, BUN and plasma creatinine (Fig. 2A–C). However, no benefits were provided by FIBRO EVs (Fig. 2A–C). Moreover, urine volume and water up-take, which were markedly increased in STZ-induced diabetic mice, were restored in stem cell-treated, but not in FIBRO EV-treated mice (Fig. 2D,E).

### EVs released by HLSCs and MSCs, but not by fibroblasts, attenuate renal histopathological changes

**Figure 3:**
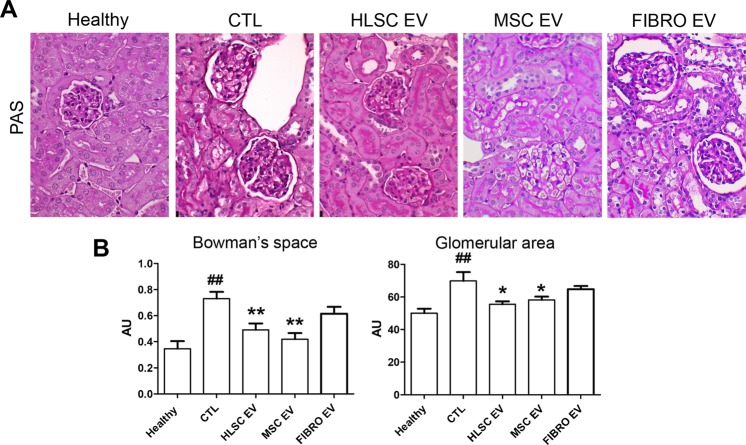
HLSC and MSC EVs ameliorated glomerular alterations in diabetic nephropathy. (**A**) Representative images of PAS-stained sections of healthy, STZ-diabetic mice (CTL) and EV-treated mice, analyzed 60 days (T60) after the onset of diabetes (magnification: 400X). (**B**) Quantification of Bowman’s space and glomerular area of healthy, CTL and EV-treated mice. Data are expressed in AU (arbitrary units) as mean ± SEM; ^##^p < 0.001 versus healthy; *p < 0.05 and **p < 0.001 versus CTL (n = 8). ANOVA with Dunnet’s multi comparison test was performed. No significant differences were observed between CTL and FIBRO EV.

**Figure 4:**
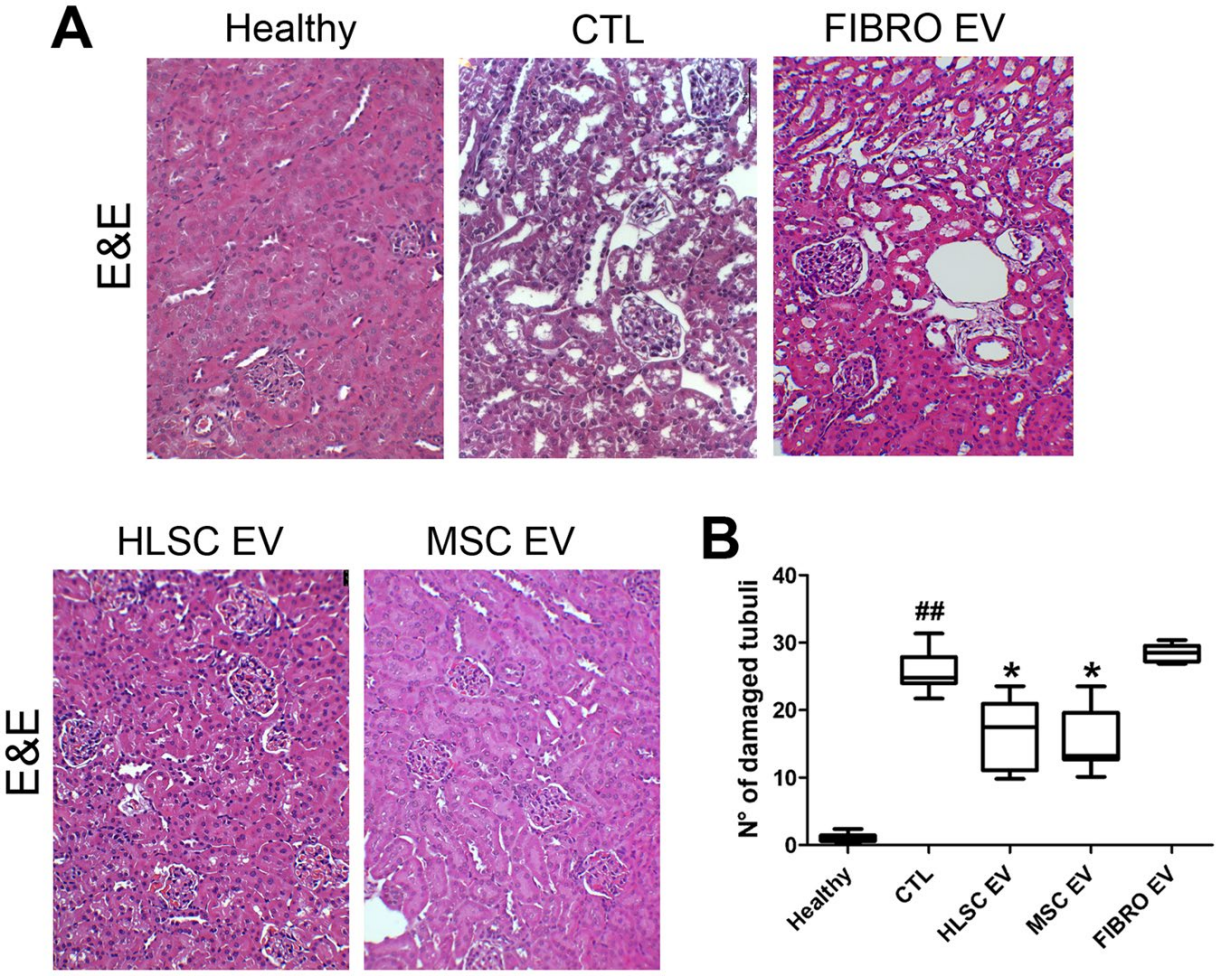
HLSC and MSC EVs reduced tubular damage in diabetic nephropathy. (**A**) Representative images of hematoxylin and eosin stained sections of healthy, STZ-diabetic mice (CTL) and EV-treated mice, analyzed 60 days (T60) after the onset of diabetes (magnification: 200X). (**B**) Quantification of tubular damage in healthy, CTL and EV-treated mice. Data are expressed as number of damaged tubuli/HPF. Data are expressed as mean ± SEM; ^##^p < 0.001 versus healthy; *p < 0.05 versus CTL (n = 8). ANOVA with Dunnet’s multi comparison test was performed. No significant differences were observed with CTL and FIBRO EV.

The beneficial effects displayed by stem cell-derived EV administration on renal function parameters were also supported by morphological analyses. Kidneys from DN mice showed different abnormalities on day 60 from the onset of diabetes. Glomeruli were hypertrophic and displayed basement-membrane thickening and an increase in Bowman’s space, as revealed by PAS staining (Fig. 3A). These pathological alterations were restored by stem cell-derived EV treatment (Fig. 3). The morphometric quantification of Bowman’s space and glomerular area were significantly decreased, compared to STZ-diabetic mice, by HLSC and MSC EV injections (Fig. 3B). Moreover, tubular hypertrophy was evident under hyperglycemic conditions. This led to tubulointerstitial fibrosis and subsequently tubular atrophy in STZ-diabetic mice. The HLSC and MSC EV treatments significantly decrease tubular injury in STZ-diabetic mice (Fig. 4). On the other hand, morphological changes were unaffected by FIBRO EV treatment (Figs. 3 and 4).

### EVs released by HLSCs and MSCs decrease fibrosis

**Figure 5:**
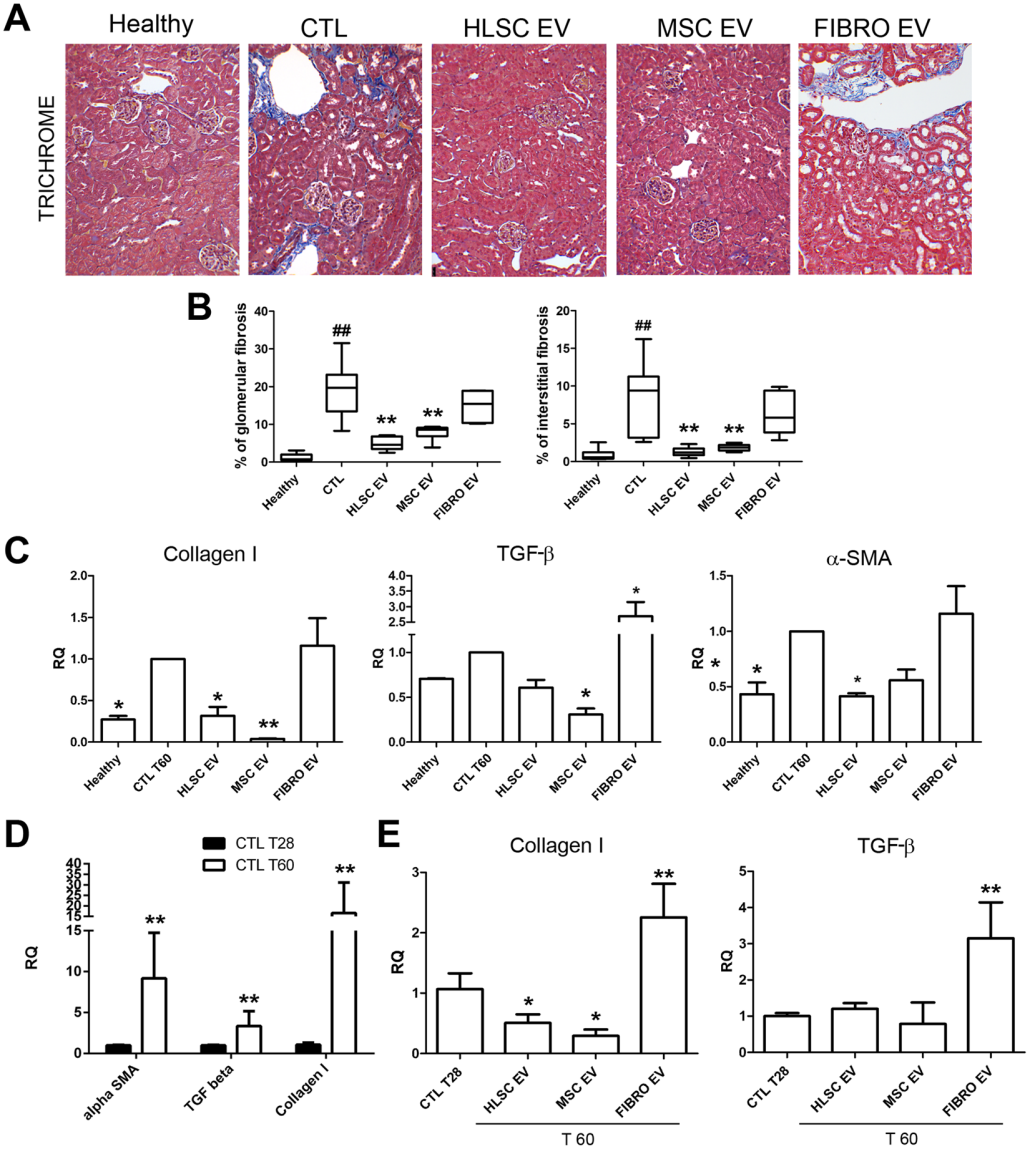
HLSC and MSC EVs improve renal fibrosis in diabetic nephropathy. (**A**) Representative images of Masson’s trichrome sections of healthy, CTL and EV-treated mice (magnification: 200X). (**B**) Quantification of glomerular and interstitial fibrosis. Data are expressed as percentage of fibrosis/glomerular area and percentage of fibrosis/total area. Data are expressed as mean ± SEM; ^##^p < 0.001 versus healthy; **p < 0.001 versus CTL (n = 8). ANOVA with Dunnet’s multi comparison test was performed. (**C**) Gene expression levels of fibrotic markers (collagen I, TGF-β and α-SMA) in mice treated with HLSC, MSC and FIBRO EVs with respect to STZ-diabetic mice (CTL), analyzed 60 days (T60) after the onset of diabetes. Data are normalized to GAPDH and to 1 for CTL, and are expressed as Relative Quantification ± SD (n = 4); *p < 0.05 and **p < 0.001 versus CTL. ANOVA with Dunnet’s multi comparison test was performed. (**D**) Gene expression levels of α-SMA, collagen I and TGF-β in STZ-diabetic mice analyzed 28 (T28) and 60 (T60) days after the onset of diabetes. Data are normalized to GAPDH and to 1 for CTL T28 and are expressed as Relative Quantification ± SD gene (n = 4); **p < 0.001 versus CTL T28. Student’s t-test was performed. (**E**) Gene expression levels of collagen I and TGF-β in mice treated with HLSC, MSC and FIBRO EVs, analyzed after 60 days (T60), with respect to STZ-diabetic mice (CTL), analyzed after 28 days (T28), starting point of EV treatment. Data are normalized to GAPDH and to 1 for CTL T28 and are expressed as Relative Quantification ± SD (n = 4); *p < 0.05 versus CTL T28. ANOVA with Dunnet’s multi comparison test was performed.

The administration of stem cell-derived EVs reduced the progression of the pro-fibrotic processes and also reverted it. The kidneys of STZ-diabetic mice, which were sacrificed on day 60 after the onset of diabetes, showed marked interstitial and glomerular fibrosis, as evaluated by trichrome staining (Fig. 5A,B). The progressive accumulation of extracellular matrix components and the activation of fibrotic pathways were both confirmed by the up-regulation of collagen I, TGF-β and α-SMA mRNAs (Fig. 5C). The quantification of renal trichrome sections showed a marked reduction in interstitial and glomerular collagen deposition upon stem cell-derived EV administration, but not after FIBRO EV injection (Fig. 5A,B). Similarly, the molecular analysis of renal tissue by quantitative real-time PCR (qRT-PCR) confirmed that at the end of experiment (T60) a decreased collagen I, TGF-β and α-SMA occurred after stem cell-derived EV treatment (Fig. 5C). On the contrary, no significant differences in collagen I, TGF-β, and α-SMA expression were detected after FIBRO EV administration (Fig. 5C). The expression of fibrotic genes get worst over time as a consequence of the continuous hyperglycemic conditions (Fig. 5D). However, when their expression levels on day 28 (starting point of EV administration) were compared with those of stem cell EV-treated mice on day 60, a significant collagen I reduction was observed. This suggests that a reversion of the fibrotic process was occurring after stem cell EV treatment (Fig. 5E). In addition, TGF-β mRNA expression did not increase in stem cell EV-treated kidneys at variance of untreated (Fig. 5E).

### Genes involved in the development of fibrosis are down-regulated by HLSC and MSC EVs

**Figure 6:**
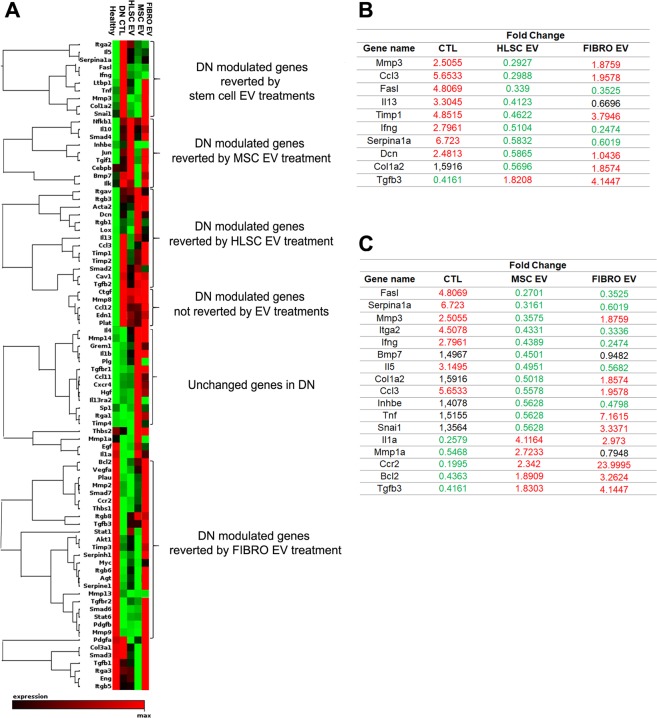
Expression of fibrosis-related genes in diabetic nephropathy. (**A**) Heatmap displaying hierarchical clustering of the entire dataset of expressed genes in the different experimental groups: healthy, STZ-diabetic mice (CTL), HLSC EV, MSC EV and FIBRO EV-treated mice. Clustering analysis showed that some groups of genes altered in DN were restored to healthy levels via treatment with one of either HLSC or MSC EVs, whereas others were restored by both stem cell EV sources (n = 3 for each experimental condition). (**B** and **C**) List of genes altered in DN and reverted by HLSC EVs (**B**) and by MSC EVs (**C**). The expression levels of reverted genes was also shown for FIBRO EV group (FIBRO EV column). Data are represented as fold change of STZ-diabetic mice with respect to healthy (CTL column) and CTL mice are compared with EV-treated mice (HLSC EV, MSC EV and FIBRO EV columns). Complete data are provided as a Supplementary Table 1.

The possibility that the protective effects of HLSC and MSC EVs depend on the regulation of genes that are associated with the induction of fibrosis was investigated. To this purpose, an RT² Profiler™ PCR Array was performed on genes involved in fibrosis in renal tissues. The gene-expression profiles of the experimental groups - healthy and diabetic mice that were either treated with EVs or left untreated - were compared. Figure 6A shows the heatmap of genes expressed in the experimental groups. Clustering analyses revealed a clear pattern for genes that were modulated in diabetic mice (Fig. 6A and Supplementary Table 1). The expression of a cluster of genes, including metalloproteinase 3 (MMP3), collagen I, tissue inhibitor of metalloproteinases (TIMP), SNAI1, chemokine (C-C motif) ligand 3 (CCL3), Serpina1, interferon γ and Fas Ligand was completely restored by both stem cell EV treatments, while others were specifically modulated by either HLSC or MSC EVs (Fig. 6A and Supplementary Table 1). In particular, a comparison of healthy and STZ-diabetic mice revealed that fifteen of the 84 genes analyzed were downregulated, while nineteen were upregulated in DN mice (Supplementary Table 1). Five of the upregulated genes were downregulated by both HLSC and MSC EV treatments, while four were modulated by HLSC EVs and seven by MSC EVs only. It is worth noting that the expression of these genes recapitulated that of the healthy group (Fig. 6B,C). The gene expression profile of FIBRO EV-treated mice revealed that the levels of the majority of genes were comparable to those of untreated STZ-diabetic mice; all the upregulated genes in STZ-diabetic mice were also increased in FIBRO EV group (Fig. 6). However, a cluster of DN modulated genes was reverted by FIBRO EV (Fig. 6A and Supplementary Table 1).

### EV miRNA cargo modulates fibrosis-related genes

**Figure 7:**
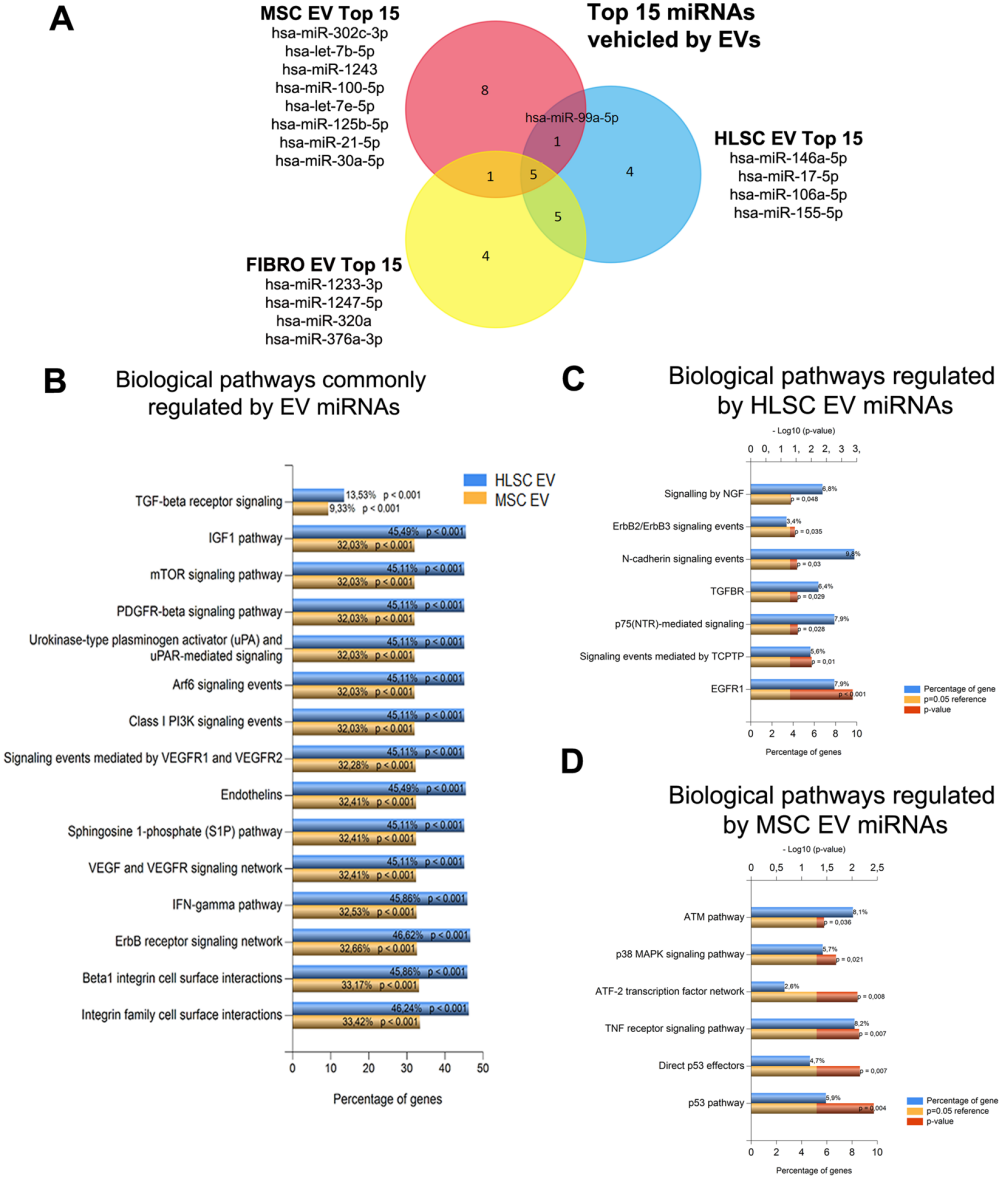
EV miRNA characterization. (**A**) Venn diagram showing the intersection between the top 15 miRNAs (See complete data as Supplementary Tables 2–4) carried by EVs from the 3 different cell sources: HLSCs, MSCs and FIBRO. (**B**) Chart representing the biological pathways commonly regulated by the miRNAs carried by HLSC EVs (blue) and by MSC EVs (yellow). The percentage of genes included in the pathway and the adjusted p-value (Hypergeometric test with Bonferroni correction) are provided. (**C** and **D**) Chart representing biological pathways specifically modulated by HLSC EVs (**C**) and MSC EVs (**D**). Blue bar represents the percentage of genes in the dataset that belong to the specific pathway; the red bar indicates the adjusted p-value for each pathway (Hypergeometric test with Bonferroni correction); the yellow bar represents the reference adjusted p-value (adj p < 0.05).

miRNA EV content has been reported to play a crucial role in the mechanisms of EV action^29,30^. The miRNA cargos of stem cell-derived EVs and FIBRO EVs were therefore compared. 700 miRNAs were analyzed for this purpose. These miRNAs were compared to those that were previously described to be present in MSC EVs by Collino *et al*.^30^. A comparison of the 15 most expressed miRNAs in each EV source (Supplementary Tables 2–4), highlighted that 5 common miRNAs were shared by all EV populations (Fig. 7A) while 4 miRNAs were expressed by HLSC EVs and 8 by MSC EVs (Fig. 7A). These miRNAs were used for predictive target analysis. 2154 genes were predicted to be targets of MSC EV miRNAs and 850 for HLSC EV miRNAs. Enrichment pathway analysis showed that these genes are involved in 52 common pathways, including the TGF-β, EGFR, PDGFR, ARF6, mTOR, and VEGF pathways (Fig. 7B). Adhesion molecule-cadherin pathways were exclusively modulated by HLSC EVs (Fig. 7C), while the p53-apoptosis, ATM and TNF pathways were associated with specific MSC EV miRNA targets (Fig. 7D).

## Discussion

The present study demonstrates that HLSC and MSC EVs inhibit the progression of DN in NSG mice that are treated with STZ. Both stem cell-derived EV types exerted beneficial effects on DN progression by restoring urinary volume and water uptake, as well as renal physiological parameters, such as ACR, BUN and plasma creatinine. The improvement in kidney morphology, the decrease in tubular and glomerular injury and the inhibition of kidney fibrosis were consistent with the functional data. In particular, stem cell-derived EVs significantly inhibited common DN features, such as glomerular thickening and the expansions of the glomerular area and Bowman’s space. Moreover, stem cell-derived EVs specifically prevented and reverted the progression of glomerular and interstitial fibrosis, as indicated by morphological and molecular analyses of the kidney. Evidence for the specific effects of stem cell-derived EV is supported by the ineffectiveness of FIBRO EVs.

Several studies have suggested that stem cell-derived EVs may play an emerging role as an alternative therapeutic approach to tissue regeneration^31^. While EVs home in injured tissues that are similar to their cell of origin^32,33^, they can also display a superior safety profile to cell-based therapy^34^. The healing properties of stem cell-derived EVs have been described in a number of pre-clinical disease models^35–37^. In particular, the therapeutic potential of MSC EVs in pre-clinical models of AKI and CKD has been reported by our own as well as other groups^23,24,28,38^. MSC EVs that were isolated from bone marrow and from cord blood have also been found to be effective in cisplatin lethal and sub-lethal models of AKI as they increased survival^38^ and protected kidneys from oxidative stress-induced apoptosis^39^. HLSC EVs have also been shown to induce recovery in a model of AKI^19^. EVs are known to be a heterogeneous population that includes vesicles that are derived from multi-vesicular bodies and cell surface budding. An attempt to identify the most active fraction in AKI protection, performed by applying differential ultracentrifugation, indicated that most of the biological activity was present in the 100 K fraction, which is enriched in exosomes, rather than in the 10 K fraction, which is enriched in shedding vesicles^40^. However, the best protective activity was obtained using the total fraction, which contained both exosomes and microvesicles that may act synergistically^40,41^.

The effect of EVs on CKD models has been poorly addressed^31^. Indeed, in order to prove the efficacy of EVs in CKD treatment, the biological effects that are exerted by EVs during the induction phase of injury have to be dissociated from those related to late fibrotic events. The preventive approach mainly recapitulates the healing properties of EV that have been observed in AKI^31^. In this regard, Jiang *et al*.^28^ have reported that urine-purified MSC EVs have a preventive effect on the development of DN. A similar preventive effect has been reported as occurring after a single EV injection by Nagaishi *et al*.^8^. The results of the present study indicate that stem cell-derived EVs that are administered in a therapeutic-like regimen may inhibit and revert the progression of the functional and morphological dysfunction caused by DN.

Extracellular matrix (ECM) protein deposition in the interstitial and glomerular space is one of the most relevant features of DN^42^. The progressive decline of renal function strongly correlates with the development of glomerular and interstitial fibrosis^43^. Morphometric analyses coupled with collagen I and α-SMA mRNA expression in the renal tissues of EV-treated animals indicated that HLSC and MSC EVs inhibit the fibrotic process and CKD progression. Moreover, observations that fibrosis and collagen I expression in renal tissues were significantly reduced in treated mice, as compared mice before treatment, suggests that partial reversal of fibrosis occurred. TGF-β expression did not increase in DN mice, which is consistent with these findings. Moreover, genes involved in the development of DN, such as Serpina1a, FAS ligand, CCL3, TIMP1, MMP3, collagen I and SNAI1, were found to be downregulated in the renal tissue of stem cell EV-treated animals. More importantly, their expression levels were comparable to those of healthy animals. The EMT plays a fundamental role in DN progression and contributes to the initial event of fibrosis. Snail (gene name: SNAI1) is considered to be an initiator of EMT^10,44^. Snail modulates tubulointerstitial fibrosis by repressing the expression of E-cadherin and by inducing the expression of α-SMA, fibronectin and collagen I in myofibroblasts^45^. We found that stem cell-derived EVs, but not FIBRO EVs, down-regulated Snail and collagen 1 expression in renal tissues. Metalloproteinases (MMPs) and tissue inhibitor of metalloproteinases (TIMP), which are proteins involved in ECM deposition/remodeling, are also dysregulated during DN progression^46,47^. The increased expression of MMP3 (also called stromelysin) and TIMP1 have been reported in a rat model of STZ-induced DN^48^. Similar up-regulation was observed in our experimental mouse model and stem cell EV, unlike FIBRO EVs, treatment completely reversed their expression. Moreover, Serpina1a (α-1-antitrypsinin), which has been proposed as a urinary and serum DN biomarker^49,50^, was found to be the most upregulated molecule in our model of DN and, once again, its expression was completely reverted by stem cell-EV administration. The FAS ligand, which is known to regulate the survival of interstitial fibroblasts in the kidneys^51^, has also been found to be upregulated in a chronic cyclosporine nephrotoxicity model^52^. In our experimental model, the expression of the Serpina1a and FAS ligand was comparable to that of healthy controls after all types of EV treatments. Finally, CCL3, which is also known as macrophage inflammatory protein 1α (MIP-1α), which is one of the most relevant chemokines in the recruitment of monocyte/macrophages and T lymphocytes during renal inflammation in humans and has been proposed as a new therapeutic target for AKI and CKD^53^, was found to be significantly downregulated in the kidneys of DN animals that were treated with stem cell-derived EVs. This was not the case for FIBRO EVs. Therefore, the anti-fibrotic effects of stem cell EVs were further supported by data obtained in animals treated with FIBRO EVs.

The RNAs carried by EVs have been shown to have a central role in EV biological activities^29,54^. EV miRNA contents that are potentially involved in the recovery of AKI have previously been characterized^30,37^. A specific miRNA signature was detected for HLSC and MSC EVs by taking into consideration the most abundant miRNAs that are differentially expressed by stem cells and FIBRO EVs. Despite differences in miRNA expression in HLSC and MSC EVs, bioinformatics analyses have indicated that they may act on common targets and on similar pro-fibrotic pathways, such as TGF-β, IGF-1, EGFR and PDGFR^16,25,40,41^, which is consistent with their therapeutic effects. The following miRNAs, miRNA-29a, let-7 family, miRNA-30a, miRNA-24 and miRNA-21, which are enriched in HLSC and MSC EVs, are known to directly target Collagen I^55,56^, Snail^57^ and the FAS ligand^58^. Interestingly, miR-146a, which has been observed to be involved in the inhibition of inflammation and fibrosis in CKD and proposed as a synthetic drug for renal fibrosis in a UUO model^59,60^, was enriched in HLSC EVs. Accordingly, let-7 and miR-30 family members that have a role in renal regeneration^18,42^ were enriched in MSC EVs.

In conclusion, our data indicate that multiple injections of stem cell-derived EVs improved renal function by preventing and partially reverting fibrosis in DN. Moreover, bioinformatic analyses indicate that EVs are enriched in miRNAs, some of which are common and others specific, that target the biological pathways involved in the development of fibrotic processes, which is consistent with the anti-fibrotic effects exerted by EVs in DN animals.

## Materials and Methods

### Cell Culture

HLSCs were isolated from human cryopreserved normal adult hepatocytes (Lonza, Basel, CH) as previously described^26^. Cells were cultured in α-MEM/EBM-1 (3:1) (Lonza) media supplemented with l-glutamine (5 mM), penicillin (50 IU/ml), streptomycin (50 μg/ml) (all from Sigma, St. Louis, MO, USA) and 10% heat-inactivated fetal calf serum (FCS) (EuroClone, Milan, IT). The presence of mesenchymal stem cell markers and the absence of endothelial and hematopoietic markers were both confirmed using cytofluorimetric analyses, as previously described^26^ (not shown). Human bone marrow MSCs were purchased from Lonza, and were cultured and characterized as previously described^16^. Cells were used within seven passages and the characterization was performed via cytofluorimetric analysis for the expression of typical mesenchymal markers, as previously described^16^. The human lung fibroblast cell line MRC5 PD 19 was obtained from Sigma.

### Isolation and characterization of EVs

EVs were obtained from the supernatants of HLSCs, MSCs and fibroblasts that were cultured overnight in RPMI without FCS. After the removal of cell debris and apoptotic bodies, via centrifugation at 3,000 g for 15 minutes and microfiltration over a 0.22 μm filter (Steroglass, PG, Italy), EVs were purified by 2 hours of ultracentrifugation at 100,000 g and 4 °C (Beckman Coulter Optima L-90 K ultracentrifuge; Beckman Coulter, Fullerton, CA). EVs were resuspended in RPMI medium (EuroClone) that contained 1% of DMSO (Sigma) and stored at −80 °C. Particle size and concentration were measured using a NanoSight NS300 (NanoSight Ltd., Amesbury, UK) equipped with a 405 nm laser, and analyzed using Nanoparticle Tracking Analysis (NTA) 3.2 software. The mean EV diameter was approximately 162 ± 59 nm.

### Mouse model of diabetic nephropathy

Animal studies were conducted in accordance with National Institute of Health Guidelines for the Care and Use of Laboratory Animals. All procedures were approved by the Ethics Committee of the University of Turin and the Italian Health Ministry (authorization number: 280/2016-PR). Eight-week-old male NSG mice were purchased from the animal facility at the Molecular Biotechnology Centre. Diabetes was induced via the intraperitoneal injection of STZ (37 mg/kg) that had been dissolved in freshly made 0.1 mol/l citrate buffer, at pH 4.5, for 4 consecutive days in order to avoid acute STZ toxicity, according to Animal Models of Diabetic Complications Consortium guidelines (available at http://www.amdcc.org)^61^.

Blood glucose levels were measured, after 4 hours of fasting, in blood from the tail-vein using a blood glucometer (GlucoMen LX Plus+, A. Menarini diagnostics, Florence, IT). The onset of diabetes was established by measuring glycaemia (up to 250 mg/ml) 10 days after STZ injection (T0). Glycaemia was monitored every 2 weeks and body weight and water up-take every week. After one month of diabetes, mice were randomly divided into the following groups: the HLSC EV, MSC EV and FIBRO EV groups, which were injected intravenously with 1 × 10^10^ particles each injection, once a week from day 30 (T30) for 4 weeks (5 injections) and the CTL group, which was injected with an equal volume of saline. Mice were sacrificed either 28 or 60 days post diabetes. At either day 60 (T60) or day 28 (T28), urine (12 hours collection in metabolic cage) and blood were collected for the evaluation of albuminuria, creatinuria, plasma creatinine and BUN. Kidneys were collected for histology and molecular analyses.

### Physiological and histological analyses

Urine samples were collected overnight on the day prior to sacrifice and albuminuria concentration was determined using an Albumin Mouse ELISA Kit (Abcam, Cambridge, UK) according to the manufacturer’s protocols. The urinary albumin results were normalized to urinary creatinine levels, which were determined using a colorimetric assay that was based on the Jaffé method (QuantiChrom Creatinine Assay Kit, BioAssay System, Hayward, USA), and were expressed as ACR.

Plasma was used to measure creatinine concentration, using a Creatinine Assay kit (Abcam), while BUN was measured using a Urea Nitrogen Colorimetric Detection kit (Arbor Assays, Michigan, USA) according to the manufacturers’ protocols.

Renal tissues were embedded in paraffin and 5 µm sections were stained with hematoxylin and eosin staining, as well as PAS and Masson’s Trichrome staining (Bio-Optica, Milan, IT) according to the manufacturer’s protocols. Interstitial and glomerular fibrosis were quantified in Masson’s Trichrome stained sections, using ImageJ software, calculated from 10 random cortical pictures per section at 200X magnification, and expressed as mean values ± SEM^60,62^. Glomerular injury was determined by measuring glomerular area and Bowman’s space using ImageJ software on 15 glomeruli from each mouse that were stained with PAS (magnification: 400X)^59,62^. The number of injured tubules in 10 random images per cortical section was counted using 200X magnification with hematoxylin and eosin staining.

### Transcriptomic analysis

The kidneys of healthy, CTL, HLSC, MSC and FIBRO EV-treated mice were homogenized in a Bullet blender (Next Advance Inc, NY, USA) at a speed of 8 rpm for 3 minutes using 3.2 mm size zirconium pellets. RNA was extracted using Trizol (Ambion, Austin, TX, USA), according manufacturer’s instructions, and quantified on a Nanodrop ND-1000 (Nanodrop, Wilmington, DE, USA) spectrophotometer. Four hundred nanograms of RNA were retrotranscribed for each sample using a RT^2^ First Strand kit (Qiagen, Frederick, MD, USA) according the manufacturer’s instructions. cDNA was run using a RT² Profiler™ PCR Array Mouse Fibrosis- PAMM-120Z **(**Qiagen) on a StepOne PlusTM System (Applied Biosystems, Foster City, CA, USA). Data analysis was conducted using Expression Suite software (Applied Biosystems) with appropriate threshold and baseline values. The Ct cut-off was set at <35. Differential expression analysis was performed using Qiagen on-line software and by comparing the expression of all groups versus CTL mice and versus healthy mice. We selected and considered the geometric mean of housekeeping genes with low variability for normalization among the different samples. Relative quantification (RQ) was calculated using the ΔΔCt method. Genes with RQ < 0.6 were considered to be downregulated (green) and ones with RQ > 1.8 were considered to be upregulated (red). p values were calculated using a Student’s t-test of the replicate 2^−ΔCt^ values for each gene in the control and treatment groups, and was set at <0.05).

The expression of the pro-fibrotic markers α-SMA, Collagen I and TGF-β was evaluated using qRT-PCR. cDNA was obtained using a High Capacity cDNA reverse transcription kit (Applied Biosystems) and PCR reactions were carried out using a Power SYBR Green PCR Master Mix (Applied Biosystems) and the specific set of primers (the complete list of primer sequences was provided in Supplementary Table 5). Mouse GAPDH was used as the housekeeping gene. Data were analyzed according to the ΔΔCt method.

### miRNome analysis

HLSC and FIBRO EVs, derived from three different preparations, were used for the extraction of RNA by a mirVana RNA isolation kit (Ambion), according to the manufacturer’s protocol. miRNA content was analyzed using the qRT-PCR method and the Applied Biosystems TaqMan™ Array Human MicroRNA A + B Cards Set v3.0 (Applied Biosystems), run on QuantStudio 12K Flex (Applied Biosystems). Briefly, 140 nanograms of RNA were reverse transcribed with a Megaplex RT Pools kit (Applied Biosystems), according to the manufacturer’s protocol. TaqMan® PreAmp Master Mix 2X (Applied Biosystems) and specific Megaplex™ PreAmp Primers (10X) (Applied Biosystems) were used to pre-amplify each cDNA sample, which were then loaded onto the TaqMan MicroRNA Array. Raw Ct values, the automatic baseline, threshold and comparison of miRNA expression were analyzed using Expression Suite software (Thermo Fisher Scientific, Waltham, MA). Data were matched with the MSC EV miRNA expression dataset, published in Collino *et al*.^30^.

Pathway enrichment analysis was performed using Funrich V3.1.3 software^63^, and data with an adjusted p-value < 0.05 were considered to be statistically significant (Hypergeometric test with Boferroni correction).

### Statistical analysis

Statistical analyses were performed using Graph Pad Prism version 5.04 (Graph Pad Software, Inc, La Jolla, CA, USA). Comparisons between groups were either analyzed using a Student’s t-test or by ANOVA, followed by the Dunnet’s multi comparison test, when appropriate. A p value of <0.05 was considered significant.

## Supplementary information

Supplemental Info
