# Supplementary material for: Stem cell-derived extracellular vesicles inhibit and revert fibrosis progression in a mouse model of diabetic nephropathy
Source: Sci Rep. 2019 Mar 14;9:4468. doi: 10.1038/s41598-019-41100-9 (PMC6418239; doi:10.1038/s41598-019-41100-9)
Supplement: Supplementary file 1 — Supplemental Info [file 41598_2019_41100_MOESM1_ESM.pdf]

Stem cell-derived extracellular vesicles inhibit and revert fibrosis progression in a mouse model of diabetic nephropathy

Cristina Grange[1,2], Stefania Tritta[2], Marta Tapparo[1], Massimo Cedrino[2], Ciro Tetta[3], Giovanni Camussi[1,2,4*] and Maria Felice Brizzi[1,2,4*]

[1] Department of Medical Sciences, University of Turin, Turin, Italy, [2] Molecular Biotechnology Centre, University of Turin, Turin, Italy, [3] Unicyte Srl, Turin, Italy, [4] 2i3T Società per la gestione dell'incubatore di imprese e per il trasferimento tecnologico Scarl, University of Turin, Turin, Italy,

Supplementary material

Supplementary Table S1. Fibrosis PCR array.

Position	Symbol	Fold Change (comparing to CTL group)											
		Healthy			HLSC EV			MSC EV			FIBRO EV		
		Fold Change	95% CI	p value	Fold Change	95% CI	p value	Fold Change	95% CI	p value	Fold Change	95% CI	p value
A01	Acta2	0.713	(0.33. 1.10)	0.286	0.946	(0.55. 1.35)	0.692	1.306	(0.70. 1.92)	0.335	1.701	(0.78. 2.63)	0.1309
A02	Agt	2.321	(0.00001. 5.07)	0.17562	1.460	(0.00001. 4.07)	0.505	0.912	(0.00001. 2.52)	0.866	3.379	(0.00001. 7.31)	0.0403
A03	Akt1	1.572	(0.57. 2.58)	0.20529	1.196	(0.00001. 2.65)	0.548	0.868	(0.00001. 1.90)	0.891	2.324	(0.86. 3.78)	0.0422
A04	Bcl2	2.291	(0.00001. 4.70)	0.12006	1.356	(0.00001. 3.28)	0.610	1.890	(0.00001. 3.83)	0.237	3.262	(0.00001. 6.63)	0.0178
A05	Bmp7	0.668	(0.55. 0.78)	0.01233	1.038	(0.83. 1.24)	0.710	0.450	(0.17. 0.73)	0.045	0.948	(0.78. 1.11)	0.5867
A06	Cav1	0.576	(0.27. 0.88)	0.16408	0.804	(0.34. 1.27)	0.4659	1.031	(0.40. 1.66)	0.978	0.999	(0.46. 1.54)	0.8522
A07	Ccl11	0.233	(0.00001. 0.50)	0.10707	1.165	(0.08. 2.25)	0.9738	4.446	(0.15. 8.74)	0.029	3.013	(0.27. 5.75)	0.0421
A08	Ccl12	0.389	(0.03. 0.75)	0.15181	0.79	(0.00001. 2.42)	0.6169	0.808	(0.00001. 2.36)	0.664	12.066	(0.00001. 57.22)	0.2399
A09	Ccl3	0.176	(0.04. 0.32)	0.05148	0.298	(0.10. 0.50)	0.0733	0.557	(0.17. 0.95)	0.1895	1.957	(0.00001. 4.10)	0.3414
A10	Ccr2	5.012	(0.32. 9.70)	0.16474	1.367	(0.00001. 3.73)	0.4624	2.342	(0.26. 4.43)	0.2396	23.999	(5.28. 42.71)	0.0673
A11	Cebpb	1.028	(0.40. 1.65)	0.97857	1.331	(0.00001. 3.33)	0.4613	0.533	(0.00001. 1.37)	0.9140	2.372	(1.05. 3.70)	0.0305
A12	Col1a2	0.628	(0.01. 1.25)	0.31593	0.569	(0.00001. 1.27)	0.3978	0.501	(0.00001. 1.06)	0.2700	1.857	(0.00001. 4.86)	0.4312
B01	Col3a1	0.986	(0.54. 1.44)	0.89048	0.777	(0.18. 1.38)	0.6634	0.594	(0.29. 0.90)	0.1281	7.040	(0.00001. 19.98)	0.3061
B02	Ctgf	0.661	(0.49. 0.83)	0.03714	0.979	(0.48. 1.48)	0.9239	0.991	(0.50. 1.48)	0.8933	1.392	(0.80. 1.99)	0.1831
B03	Cxcr4	0.664	(0.34. 0.99)	0.19305	1.042	(0.00001. 2.45)	0.5998	2.300	(0.92. 3.68)	0.0737	1.827	(1.26. 2.40)	0.0073
B04	Dcn	0.403	(0.16. 0.64)	0.06774	0.586	(0.20. 0.98)	0.1640	1.482	(0.52. 2.45)	0.2895	1.043	(0.05. 2.04)	0.8359
B05	Edn1	0.2273	(0.16. 0.30)	0.00489	0.8226	(0.40. 1.24)	0.5743	0.724	(0.27. 1.18)	0.3640	0.909	(0.22. 1.60)	0.9504
B06	Egf	1.3471	(0.79. 1.90)	0.21594	1.034	(0.22. 1.85)	0.7530	1.310	(0.43. 2.20)	0.4408	1.119	(0.71. 1.52)	0.6075
B07	Eng	1.193	(0.95. 1.44)	0.14871	1.04	(0.48. 1.60)	0.7461	0.7567	(0.07. 1.45)	0.8982	1.977	(1.43. 2.52)	0.0090
B08	Fasl	0.208	(0.05. 0.36)	0.04863	0.339	(0.00001. 0.86)	0.3052	0.2701	(0.00001. 0.56)	0.0902	0.352	(0.00001. 0.77)	0.1341
B09	Grem1	0.244	(0.03. 0.46)	0.07599	3.0694	(0.00001. 8.81)	0.2082	8.6182	(2.37. 14.86)	0.0029	5.8345	(0.00001. 11.88)	0.1112
B10	Hgf	0.766	(0.18. 1.35)	0.48858	0.9323	(0.36. 1.50)	0.6367	2.3306	(0.86. 3.80)	0.0284	2.0064	(0.37. 3.64)	0.2046
B11	Ifng	0.357	(0.20. 0.52)	0.03847	0.5104	(0.30. 0.72)	0.0760	0.4389	(0.12. 0.76)	0.1134	0.2474	(0.15. 0.35)	0.0227
B12	Il10	0.244	(0.10. 0.39)	0.03062	1.4955	(0.00001. 4.44)	0.4384	0.9153	(0.00001. 2.13)	0.7792	1.2825	(0.66. 1.90)	0.353
C01	Il13	0.302	(0.03. 0.58)	0.14247	0.4123	(0.00001. 0.98)	0.3108	0.8957	(0.02. 1.77)	0.6439	0.6696	(0.00001. 1.46)	0.5245
C02	Il13ra2	0.598	(0.40. 0.80)	0.05645	0.831	(0.38. 1.28)	0.6293	2.7993	(0.05. 5.55)	0.1157	0.5476	(0.26. 0.83)	0.0840
C03	Il1a	3.877	(0.00001. 7.98)	0.07228	0.9632	(0.00001. 2.14)	0.8348	4.1164	(0.00001. 8.27)	0.0369	2.973	(0.00001. 6.43)	0.2045
C04	Il1b	0.819	(0.44. 1.20)	0.4002	1.5311	(0.00001. 3.34)	0.4373	2.2948	(1.19. 3.40)	0.0338	9.1849	(3.59. 14.78)	0.0170

Position	Symbol	Fold Change (comparing to CTL group)											
		Healthy			HLSC EV			MSC EV			FIBRO EV		
		Fold Change	95% CI	p value	Fold Change	95% CI	p value	Fold Change	95% CI	p value	Fold Change	95% CI	p value
C05	Il4	1.063	(0.09. 2.04)	0.68096	1.55	(0.46. 2.64)	0.2375	2.0454	(0.00001. 5.03)	0.3087	2.6219	(0.19. 5.05)	0.1817
C06	Il5	0.317	(0.00001, 0.68)	0,05328	0,6708	(0.00001, 1.38)	0,72942	0,4951	(0.34, 0.65)	0,0051	0,5682	(0.00001, 1.57)	0,9551
C07	Ilk	0.959	(0.65, 1.27)	0,73538	0,9866	(0.41, 1.56)	0,94185	0,9298	(0.37, 1.49)	0,9291	1,1644	(0.79, 1.54)	0,4662
C08	Inhbe	0.710	(0.29, 1.13)	0,26770	1,6969	(0.00001, 4.86)	0,42681	0,5628	(0.24, 0.89)	0,1346	0,4798	(0.20, 0.76)	0,0974
C09	Itga1	1.027	(0.66, 1.39)	0,91357	1,0106	(0.67, 1.36)	0,99555	1,4709	(1.02, 1.92)	0,0566	1,3873	(0.91, 1.87)	0,1355
C10	Itga2	0.221	(0.19, 0.26)	0,00026	0,7942	(0.57, 1.01)	0,17019	0,4331	(0.18, 0.69)	0,0300	0,3336	(0.09, 0.58)	0,0112
C11	Itga3	1.194	(0.77, 1.62)	0,43649	0,9699	(0.26, 1.68)	0,89628	0,5413	(0.00001, 1.11)	0,5065	1,8794	(0.99, 2.77)	0,0873
C12	Itgav	0.843	(0.48, 1.21)	0,42667	1,0221	(0.59, 1.45)	0,99838	1,0793	(0.65, 1.51)	0,8002	0,9762	(0.52, 1.43)	0,8813
D01	Itgb1	0,836	(0.63, 1.04)	0,22612	0,9375	(0.49, 1.38)	0,94216	1,3423	(1.05, 1.64)	0,0570	0,9864	(0.69, 1.29)	0,9767
D02	Itgb3	0,714	(0.24, 1.19)	0,41453	0,9701	(0.28, 1.66)	0,95655	1,1667	(0.55, 1.78)	0,7056	1,281	(0.50, 2.06)	0,5104
D03	Itgb5	1,153	(0.61, 1.70)	0,63367	1,0058	(0.13, 1.88)	0,82159	0,8313	(0.09, 1.57)	0,8966	1,6763	(0.69, 2.66)	0,1667
D04	Itgb6	1,786	(1.51, 2.07)	0,00185	1,3456	(0.84, 1.85)	0,22158	0,9774	(0.85, 1.10)	0,7082	1,9916	(1.58, 2.40)	0,0041
D05	Itgb8	1,671	(0.99, 2.35)	0,07472	1,4057	(0.92, 1.89)	0,12277	1,6522	(1.14, 2.16)	0,0216	1,6199	(1.04, 2.20)	0,0561
D06	Jun	0,518	(0.24, 0.79)	0,08916	1,2113	(0.00001, 2.91)	0,53108	0,6142	(0.00001, 1.40)	0,8547	1,2604	(0.67, 1.86)	0,4164
D07	Lox	0,673	(0.10, 1.25)	0,34783	0,6637	(0.00001, 1.34)	0,44585	1,6332	(0.24, 3.03)	0,3540	1,1003	(0.00001, 2.42)	0,7871
D08	Ltbp1	0,637	(0.44, 0.84)	0,04588	0,7773	(0.51, 1.04)	0,24495	0,6054	(0.10, 1.11)	0,3741	1,1741	(0.40, 1.95)	0,5465
D09	Mmp13	1,692	(0.74, 2.64)	0,18812	0,8302	(0.15, 1.52)	0,98744	0,8602	(0.08, 1.64)	0,9463	0,8741	(0.23, 1.52)	0,9847
D10	Mmp14	1,076	(0.37, 1.79)	0,93222	1,2538	(0.17, 2.34)	0,60113	1,5483	(0.17, 2.93)	0,3941	3,4191	(0.52, 6.32)	0,1172
D11	Mmp1a	1,828	(1.15, 2.51)	0,03494	1,0633	(0.00001, 2.16)	0,64523	2,7233	(1.60, 3.85)	0,0144	0,7948	(0.56, 1.03)	0,1992
D12	Mmp2	1,236	(0.33, 2.15)	0,75195	0,9535	(0.21, 1.70)	0,71717	1,0391	(0.00001, 2.08)	0,9286	3,6789	(0.00001, 8.14)	0,2142
E01	Mmp3	0,3991	(0.00001, 0.97)	0,28724	0,2927	(0.00001, 0.72)	0,25219	0,3575	(0.00001, 0.96)	0,3073	1,8759	(0.00001, 6.29)	0,4553
E02	Mmp8	0,1853	(0.00001, 0.42)	0,176161	0,8625	(0.00001, 1.90)	0,59818	0,9938	(0.00001, 2.12)	0,6790	1,1328	(0.00001, 2.60)	0,9761
E03	Mmp9	10,3285	(0.00001, 85.91)	0,373957	0,7143	(0.29, 1.14)	0,37321	0,3082	(0.00001, 0.66)	0,1001	1,6651	(0.48, 2.85)	0,2514
E04	Myc	1,7714	(0.44, 3.11)	0,179481	1,2625	(0.00001, 2.95)	0,53434	1,0175	(0.00001, 2.24)	0,7388	1,4194	(0.53, 2.31)	0,3241
E05	Nfkb1	0,8573	(0.38, 1.34)	0,488434	1,0451	(0.23, 1.86)	0,90896	0,9984	(0.29, 1.71)	0,9477	2,0547	(0.73, 3.38)	0,0824
E06	Pdgfa	0,985	(0.31, 1.66)	0,746595	0,831	(0.00001, 1.70)	0,82345	0,8988	(0.22, 1.57)	0,6563	1,1977	(0.36, 2.04)	0,7809
E07	Pdgfb	3,2063	(0.64, 5.77)	0,015168	1,1315	(0.00001, 2.50)	0,78414	1,0365	(0.18, 1.90)	0,8176	6,9448	(1.14, 12.75)	0,0051
E08	Plat	0,4922	(0.25, 0.73)	0,073591	0,8623	(0.25, 1.48)	0,81606	0,8143	(0.17, 1.45)	0,7850	1,2798	(0.69, 1.87)	0,3917

Position	Symbol	Fold Change (comparing to CTL group)											
		Healthy			HLSC EV			MSC EV			FIBRO EV		
		Fold Change	95% CI	p value	Fold Change	95% CI	p value	Fold Change	95% CI	p value	Fold Change	95% CI	p value
E09	Plau	2,7043	(0.98, 4.43)	0,035169	0,8683	(0.14, 1.59)	0,77737	1,4791	(0.55, 2.41)	0,2924	2,5798	(0.52, 4.64)	0,1472
E10	Plg	0,8329	(0.00001, 1.77)	0,698655	1,4698	(0.00001, 3.54)	0,48229	2,3092	(0.00001, 5.16)	0,1924	0,2773	(0.00001, 0.63)	0,1259
E11	Serpina 1a	0,1487	(0.07, 0.22)	0,00197	0,5832	(0.00001, 1.19)	0,62467	0,3161	(0.00001, 0.72)	0,0604	0,6019	(0.20, 1.00)	0,1837
E12	Serpine 1	2,1788	(0.00001, 5.15)	0,624232	1,408	(0.00001, 4.29)	0,70692	0,8436	(0.00001, 2.04)	0,5576	22,8945	(0.00001, 58.02)	0,0972
F01	Serpin h1	2,218	(1.58, 2.85)	0,006788	1,2359	(0.48, 1.99)	0,45933	0,9149	(0.35, 1.48)	0,9744	6,6189	(4.75, 8.49)	0,0009
F02	Smad2	0,638	(0.41, 0.86)	0,066198	0,8351	(0.47, 1.20)	0,46879	0,9316	(0.64, 1.23)	0,6187	0,7605	(0.49, 1.04)	0,2059
F03	Smad3	0,9891	(0.29, 1.69)	0,780635	0,7833	(0.00001, 1.71)	0,78857	0,6437	(0.04, 1.25)	0,445	1,8707	(0.50, 3.24)	0,1688
F04	Smad4	0,7603	(0.42, 1.10)	0,300751	1,1339	(0.61, 1.66)	0,73045	0,9305	(0.46, 1.40)	0,7247	1,7013	(0.91, 2.49)	0,0829
F05	Smad6	1,6824	(0.17, 3.19)	0,294082	1,0458	(0.00001, 2.76)	0,67621	1,0783	(0.00001, 2.48)	0,7899	3,1886	(0.32, 6.05)	0,0265
F06	Smad7	1,7182	(0.00001, 3.89)	0,491264	0,8729	(0.00001, 2.94)	0,65329	1,1466	(0.00001, 2.74)	0,9357	2,5466	(0.00001, 5.58)	0,1142
F07	Snai1	0,7373	(0.21, 1.26)	0,403976	0,6451	(0.15, 1.14)	0,28557	0,5628	(0.24, 0.89)	0,1346	3,3371	(1.26, 5.41)	0,0195
F08	Sp1	1,2819	(0.64, 1.93)	0,448666	1,1588	(0.00001, 2.36)	0,60043	2,1122	(1.08, 3.14)	0,0193	1,3672	(0.63, 2.10)	0,3442
F09	Stat1	1,1842	(0.76, 1.61)	0,479035	1,1468	(0.45, 1.84)	0,63316	1,0103	(0.64, 1.38)	0,9165	3,3915	(0.00001, 7.08)	0,2388
F10	Stat6	3,2018	(2.24, 4.16)	0,007051	1,3274	(0.52, 2.14)	0,31317	1,3639	(0.32, 2.41)	0,3954	8,297	(5.89, 10.70)	0,0021
F11	Tgfb1	1,1988	(0.91, 1.49)	0,220366	0,9129	(0.37, 1.45)	0,94414	0,6878	(0.13, 1.25)	0,6389	2,9634	(1.52, 4.40)	0,0395
F12	Tgfb2	0,426	(0.17, 0.68)	0,089949	0,74	(0.10, 1.38)	0,60346	0,9646	(0.29, 1.63)	0,8843	1,0023	(0.35, 1.65)	0,9508
G01	Tgfb3	2,4031	(0.84, 3.97)	0,056296	1,8208	(0.14, 3.51)	0,21319	1,8303	(0.73, 2.93)	0,1116	4,1447	(1.63, 6.65)	0,0125
G02	Tgfbr1	0,9517	(0.46, 1.44)	0,670594	1,0787	(0.15, 2.00)	0,76604	1,8046	(0.83, 2.78)	0,0623	1,7895	(0.68, 2.90)	0,1268
G03	Tgfbr2	1,8015	(0.00001, 3.63)	0,289131	1,2371	(0.00001, 3.27)	0,59598	1,1509	(0.00001, 2.78)	0,7524	3,4309	(0.00001, 7.21)	0,0895
G04	Tgif1	0,6344	(0.00001, 1.39)	0,330002	1,2778	(0.00001, 3.01)	0,78503	0,7567	(0.00001, 1.70)	0,5022	1,4982	(0.00001, 3.18)	0,6778
G05	Thbs1	1,6934	(0.95, 2.43)	0,084373	1,0511	(0.17, 1.93)	0,72231	1,293	(0.79, 1.79)	0,2797	3,6959	(1.55, 5.84)	0,0495
G06	Thbs2	1,1139	(0.95, 1.28)	0,215155	0,6943	(0.14, 1.25)	0,46881	1,2515	(0.94, 1.56)	0,1611	1,6755	(0.85, 2.50)	0,1658
G07	Timp1	0,2061	(0.00001, 0.43)	0,155145	0,4622	(0.00001, 0.97)	0,26750	0,7535	(0.00001, 1.54)	0,4631	3,7946	(0.00001, 10.33)	0,3283
G08	Timp2	0,6423	(0.36, 0.92)	0,117676	0,7633	(0.24, 1.28)	0,54099	0,8821	(0.19, 1.57)	0,9148	1,2575	(0.40, 2.11)	0,4952
G09	Timp3	1,3917	(1.17, 1.61)	0,010804	1,119	(0.57, 1.67)	0,56697	0,8694	(0.28, 1.46)	0,9381	1,3326	(1.02, 1.65)	0,0877
G10	Timp4	1,035	(0.07, 2.00)	0,937503	0,9796	(0.03, 1.93)	0,87406	1,7996	(0.37, 3.22)	0,2138	1,3031	(0.29, 2.31)	0,7350
G11	Tnf	0,6599	(0.28, 1.04)	0,203261	0,7165	(0.12, 1.31)	0,43084	0,5628	(0.24, 0.89)	0,1346	7,1615	(0.00001, 14.61)	0,1166
G12	Vegfa	1,3985	(1.03, 1.76)	0,070221	1,0572	(0.37, 1.74)	0,68212	1,2326	(0.83, 1.64)	0,2901	1,6958	(1.28, 2.11)	0,0181

Supplementary Table S1. List of genes involved in fibrosis, screened using PCR array. Gene expression was evaluated in kidneys of healthy mice and of DN mice treated with HLSC, MSC and FIBRO EVs. Fold Change was calculated using ΔΔCt method. Genes with RQ <0.6 were considered downregulated and the one with RQ >1.8 were considered upregulated. p values were calculated based on a Student's t-test of the replicate $2^{-\Delta Ct}$ values for each gene in the control and treatment groups and was set at <0.05.

Supplementary Table S2: List of miRNAs carried by MSC EVs.

MSC EVs		
**miRNA name**	**CT mean**	**SD**
**hsa-miR-222-3p**	21.58	2.24
**hsa-miR-24-3p**	21.64	1.65
**hsa-miR-302c-3p**	21.87	7.23
**hsa-miR-99a-5p**	22.90	1.65
**hsa-let-7b-5p**	22.93	1.20
**hsa-miR-1243**	23.31	12.71
**hsa-miR-100-5p**	23.35	1.87
**hsa-let-7e-5p**	23.84	2.50
**hsa-miR-191-5p**	23.87	1.91
**hsa-miR-125b-5p**	24.08	2.06
**hsa-miR-21-5p**	24.15	2.85
**hsa-miR-193b-3p**	24.23	1.73
**hsa-miR-31-5p**	24.25	2.00
**hsa-miR-30a-5p**	24.28	3.07
**hsa-miR-574-3p**	24.33	1.80
hsa-miR-145-5p	24.42	2.49
hsa-miR-214-3p	24.45	1.63
hsa-miR-320a	24.65	1.67
hsa-miR-1260a	24.94	3.15
hsa-miR-197-3p	25.03	2.44
hsa-miR-30a-3p	25.04	2.55
hsa-miR-199a-3p hsa-miR-199b-3p	25.08	1.69
hsa-miR-374b-5p	25.09	2.41
hsa-miR-484	25.25	1.80
hsa-miR-221-3p	25.26	2.22
hsa-miR-409-3p	25.31	3.21
hsa-miR-186-5p	25.40	1.83
hsa-miR-30c-5p	25.42	2.10
hsa-miR-29a-3p	25.47	1.92
**MSC EVs**		
**miRNA name**	**CT mean**	**SD**
hsa-miR-138-5p	25.48	2.59
hsa-miR-99b-5p	25.70	1.50
hsa-miR-16-5p	25.71	1.83
hsa-miR-106a-5p	25.89	1.74
hsa-miR-888-5p	25.91	7.82
hsa-miR-17-5p	25.92	1.40
hsa-miR-19b-3p	26.03	1.72
hsa-miR-30b-5p	26.27	2.00
hsa-miR-26a-5p	26.39	1.70
hsa-miR-31-3p	26.49	1.78
hsa-miR-30e-3p	26.65	2.22
hsa-miR-125a-5p	26.77	1.89
hsa-miR-331-3p	26.81	2.08
hsa-miR-1270	26.88	2.43
hsa-miR-140-5p	27.06	1.89
hsa-miR-20a-5p	27.07	2.33
hsa-miR-149-5p	27.16	2.24
hsa-miR-376c-3p	27.27	2.40
hsa-miR-204-5p	27.33	3.62
hsa-miR-34a-5p	27.36	2.08
hsa-miR-152-3p	27.38	1.35
hsa-miR-193a-5p	27.49	2.23
hsa-miR-328-3p	27.55	1.38
hsa-miR-1275	27.61	3.10
hsa-miR-181a-5p	27.73	1.99
hsa-let-7d-5p	27.77	1.82
hsa-miR-126-5p	27.79	0.96
hsa-miR-25-3p	27.83	2.20
hsa-miR-143-3p	27.88	2.43
**MSC EVs**		
**miRNA name**	**CT mean**	**SD**
hsa-miR-92a-3p	27.89	2.39
hsa-miR-425-5p	27.94	1.03
hsa-miR-382-5p	28.19	3.60
hsa-miR-365a-3p hsa-miR-365b-3p	28.21	2.01
hsa-let-7g-5p	28.22	1.70
hsa-miR-342-3p	28.30	1.53
hsa-miR-28-3p	28.30	2.08
hsa-miR-376a-3p	28.33	2.04
hsa-miR-370-3p	28.36	2.46
hsa-miR-146b-5p	28.36	1.45
hsa-miR-150-5p	28.56	1.50
hsa-miR-133a-3p	28.69	2.68
hsa-miR-93-5p	28.79	1.42
hsa-miR-638	28.85	2.13
hsa-miR-532-5p	28.85	2.67
hsa-miR-485-3p	28.88	1.67
hsa-miR-132-3p	28.98	4.22
hsa-miR-539-5p	29.02	2.01
hsa-miR-10a-5p	29.10	1.31
hsa-miR-483-5p	29.13	2.15
hsa-miR-411-5p	29.15	1.88
hsa-miR-34a-3p	29.15	2.01
hsa-miR-374a-5p	29.24	2.11
hsa-miR-432-5p	29.26	3.00
hsa-miR-335-5p	29.33	1.80
hsa-miR-195-5p	29.34	1.81
hsa-miR-494-3p	29.35	1.98
hsa-miR-27a-3p	29.44	3.97
hsa-miR-495-3p	29.49	1.32
**MSC EVs**		
**miRNA name**	**CT mean**	**SD**
hsa-miR-218-5p	29.52	2.06
hsa-miR-134-5p	29.55	2.86
hsa-miR-345-5p	29.56	1.30
hsa-miR-10b-3p	29.58	2.08
hsa-miR-625-3p	29.65	1.62
hsa-miR-27b-3p	29.69	2.41
hsa-miR-335-3p	29.70	3.87
hsa-miR-486-5p	29.73	1.71
hsa-miR-339-3p	29.77	3.39
hsa-miR-210-3p	29.78	6.83
hsa-miR-193b-5p	29.80	1.28
hsa-miR-590-5p	29.85	2.56
hsa-miR-34b-3p	29.90	1.05
hsa-miR-744-5p	29.90	1.63
hsa-let-7c-5p	29.92	2.45
hsa-miR-454-3p	29.95	1.91
hsa-miR-379-5p	29.99	1.43
hsa-miR-769-5p	30.05	1.76
hsa-miR-323a-3p	30.06	2.13
hsa-miR-19a-3p	30.06	2.00
hsa-miR-137	30.12	2.23
hsa-miR-320b	30.13	2.29
hsa-miR-106b-5p	30.14	1.97
hsa-miR-224-5p	30.14	1.81
hsa-miR-222-5p	30.18	3.38
hsa-miR-20b-5p	30.30	1.79
hsa-miR-196b-5p	30.30	2.45
hsa-miR-29a-5p	30.33	3.07
hsa-miR-130a-3p	30.73	1.99

MSC EVs		
**miRNA name**	**CT mean**	**SD**
hsa-miR-136-3p	30.76	2.50
hsa-miR-1233-3p	30.77	6.22
hsa-miR-130b-3p	30.83	2.36
hsa-miR-26b-5p	30.93	2.60
hsa-miR-103a-3p	30.95	3.74
hsa-miR-151a-3p	30.99	3.15
hsa-miR-203a-3p	31.02	0.73
hsa-miR-202-3p	31.03	0.93
hsa-let-7a-5p	31.03	6.57
hsa-miR-214-5p	31.08	3.04
hsa-miR-99b-3p	31.11	0.77
hsa-miR-423-5p	31.11	1.54
hsa-miR-377-3p	31.12	10.28
hsa-miR-708-5p	31.18	3.96
hsa-miR-22-5p	31.33	4.42
hsa-miR-146a-5p	31.33	3.01
hsa-miR-493-3p	31.35	1.46
hsa-miR-324-3p	31.39	2.08
hsa-miR-452-5p	31.39	1.91
hsa-miR-1291	31.42	1.98
hsa-miR-636	31.44	3.49
hsa-miR-15b-5p	31.46	2.59
hsa-miR-543	31.47	3.49
hsa-miR-33a-3p	31.47	1.96
hsa-miR-155-5p	31.50	1.02
hsa-miR-30d-5p	31.51	3.18
hsa-miR-487b-3p	31.55	1.56
hsa-miR-129-2-3p	31.70	1.70
hsa-miR-885-5p	31.74	5.79

MSC EVs		
**miRNA name**	**CT mean**	**SD**
hsa-miR-664a-3p	31.84	1.76
hsa-miR-1208	31.84	3.29
hsa-miR-28-5p	31.87	5.43
hsa-miR-483-3p	31.89	5.59
hsa-miR-433-3p	31.93	5.39
hsa-miR-410-3p	31.94	1.53
hsa-miR-1271-5p	31.96	3.61
hsa-miR-601	31.96	1.96
hsa-miR-503-5p	31.97	5.63
hsa-miR-29b-3p	32.01	2.39
hsa-miR-184	32.09	5.32
hsa-miR-151a-5p	32.13	3.50
hsa-miR-1290	32.13	5.35
hsa-miR-572	32.22	2.53
hsa-miR-367-3p	32.24	9.07
hsa-miR-212-3p	32.33	5.17
hsa-miR-93-3p	32.33	5.23
hsa-miR-215-5p	32.35	2.73
hsa-miR-27a-5p	32.42	1.19
hsa-miR-223-3p	32.50	2.71
hsa-miR-505-3p	32.58	5.07
hsa-miR-142-3p	32.59	2.68
hsa-miR-296-5p	32.63	4.94
hsa-miR-532-3p	32.64	4.95
hsa-miR-770-5p	32.66	5.08
hsa-miR-10b-5p	32.72	5.97
hsa-miR-145-3p	32.75	2.59
hsa-miR-199a-5p	32.79	4.81
hsa-miR-324-5p	32.79	4.93

MSC EVs		
**miRNA name**	**CT mean**	**SD**
hsa-miR-660-5p	32.80	5.38
hsa-miR-140-3p	32.82	3.68
hsa-miR-338-5P	32.88	3.17
hsa-miR-200c-3p	32.88	1.70
hsa-miR-889-3p	32.91	1.44
hsa-miR-339-5p	33.00	4.89
hsa-miR-655-3p	33.03	4.70
hsa-miR-29c-3p	33.03	1.71
hsa-miR-1244	33.05	0.89
hsa-miR-21-3p	33.05	5.04
hsa-miR-125b-1-3p	33.22	3.58
hsa-miR-181a-3p	33.27	4.57
hsa-miR-301a-3p	33.37	4.43
hsa-miR-18a-5p	33.42	4.77
hsa-miR-1282	33.47	2.25
hsa-miR-148b-3p	33.48	4.49
hsa-miR-766-3p	33.54	4.35
hsa-miR-181a-2-3p	33.56	4.34
hsa-miR-451a	33.60	4.32
hsa-miR-194-5p	33.62	4.88
hsa-miR-362-5p	33.63	4.39
hsa-miR-671-3p	33.74	4.66
hsa-miR-500a-5p	33.79	4.44
hsa-miR-124-3p	33.82	4.19
hsa-miR-7-1-3p	33.84	2.57
hsa-miR-502-5p	33.85	4.14
hsa-miR-199b-5p	33.94	4.31
hsa-miR-548c-3p	33.95	1.12
hsa-miR-148a-3p	33.97	4.67

MSC EVs		
**miRNA name**	**CT mean**	**SD**
hsa-miR-424-3p	34.00	4.57
hsa-miR-27b-5p	34.22	4.32
hsa-miR-505-5p	34.26	2.53
hsa-miR-376b-3p	34.28	6.71
hsa-miR-425-3p	34.44	4.10
hsa-miR-628-5p	34.49	3.73

Supplementary Table S2. List of miRNAs carried by MSC EVs. Data are displayed as CT mean value ± SD (n=3). The 15 most expressed miRNAs, used in bioinformatics analysis, are highlighted in bold.

Supplementary Table S3. List of miRNAs vehicle by HLSC EVs.

HLSC EVs		
**miRNA name**	**CT mean**	**SD**
**hsa-miR-24-3p**	17.43	0.16
**hsa-miR-191-5p**	18.67	0.63
**hsa-miR-146a-5p**	19.28	3.36
**hsa-miR-222-3p**	19.52	0.30
**hsa-miR-31-5p**	19.66	0.78
**hsa-miR-574-3p**	20.60	0.30
**hsa-miR-484**	20.71	0.61
**hsa-miR-16-5p**	20.76	0.28
**hsa-miR-29a-3p**	21.15	1.13
**hsa-miR-17-5p**	21.32	0.03
**hsa-miR-106a-5p**	21.40	0.04
**hsa-miR-19b-3p**	21.45	0.00
**hsa-miR-409-3p**	21.45	0.54
**hsa-miR-155-5p**	21.50	0.33
**hsa-miR-99a-5p**	21.50	0.45
hsa-miR-320a	21.72	0.37
hsa-miR-193b-3p	22.10	0.31
hsa-miR-214-3p	22.17	0.67
hsa-let-7b-5p	22.19	0.18
hsa-miR-100-5p	22.23	0.41
hsa-miR-376c-3p	22.39	0.34
hsa-miR-342-3p	22.47	0.15
hsa-miR-126-3p	22.79	0.45
hsa-miR-186-5p	22.83	0.11
hsa-let-7e-5p	22.94	0.30
hsa-miR-125a-5p	23.24	0.09
hsa-miR-146b-5p	23.25	0.16
hsa-miR-374b-5p	23.33	0.16
hsa-miR-454-3p	23.33	0.53

HLSC EVs		
**miRNA name**	**CT mean**	**SD**
hsa-miR-151a-3p	23.45	0.53
hsa-miR-21-5p	23.45	0.23
hsa-miR-210-3p	23.46	0.40
hsa-miR-30a-3p	23.64	0.32
hsa-miR-935	23.68	0.41
hsa-miR-199a-3p<br>hsa-miR-199b-3p	23.74	0.56
hsa-miR-376a-3p	23.79	0.46
hsa-miR-26a-5p	23.98	0.02
hsa-miR-20a-5p	24.00	0.07
hsa-miR-145-5p	24.04	0.33
hsa-miR-99b-5p	24.06	0.05
hsa-miR-30e-3p	24.09	0.46
hsa-miR-331-3p	24.10	0.80
hsa-miR-28-3p	24.18	0.02
hsa-miR-30c-5p	24.32	0.00
hsa-miR-134-5p	24.52	0.56
hsa-miR-132-3p	24.59	0.46
hsa-miR-149-5p	24.59	0.21
hsa-miR-886-5p	24.65	0.62
hsa-miR-411-5p	24.66	0.25
hsa-miR-221-3p	24.79	0.48
hsa-miR-483-5p	24.92	0.86
hsa-miR-125b-5p	24.93	0.19
hsa-miR-31-3p	24.96	0.84
hsa-miR-218-5p	24.99	1.08
hsa-miR-181a-5p	25.00	0.50
hsa-miR-345-5p	25.04	0.20
hsa-miR-374a-5p	25.11	0.59
hsa-miR-494-3p	25.15	0.46

HLSC EVs		
**miRNA name**	**CT mean**	**SD**
hsa-let-7g-5p	25.18	0.24
hsa-miR-30b-5p	25.18	0.10
hsa-miR-197-3p	25.26	0.03
hsa-miR-99b-3p	25.34	0.33
hsa-miR-370-3p	25.40	0.75
hsa-miR-339-3p	25.48	0.62
hsa-miR-10a-5p	25.54	0.38
hsa-miR-34a-3p	25.56	1.10
hsa-miR-539-5p	25.57	0.24
hsa-miR-152-3p	25.58	0.30
hsa-miR-382-5p	25.62	0.40
hsa-miR-140-5p	25.74	0.27
hsa-miR-34a-5p	25.84	1.13
hsa-miR-886-3p	25.95	0.81
hsa-miR-19a-3p	26.00	0.27
hsa-miR-127-3p	26.05	0.56
hsa-miR-204-5p	26.10	0.20
hsa-miR-625-3p	26.12	0.14
hsa-miR-92a-3p	26.25	0.04
hsa-miR-431-5p	26.27	0.44
hsa-miR-425-5p	26.29	0.52
hsa-miR-1260a	26.34	2.64
hsa-miR-432-5p	26.35	0.29
hsa-let-7d-5p	26.36	0.05
hsa-miR-590-5p	26.38	0.66
hsa-miR-365a-3p<br>hsa-miR-365b-3p	26.40	0.17
hsa-miR-212-3p	26.45	0.47
hsa-miR-138-5p	26.47	1.21
hsa-miR-744-5p	26.49	0.55

HLSC EVs		
**miRNA name**	**CT mean**	**SD**
hsa-miR-660-5p	26.49	0.92
hsa-miR-195-5p	26.50	0.23
hsa-miR-223-3p	26.59	1.38
hsa-miR-379-5p	26.60	0.01
hsa-miR-664a-3p	26.76	0.78
hsa-miR-106b-5p	26.84	0.71
hsa-miR-7-1-3p	26.97	0.67
hsa-miR-27a-3p	26.98	0.62
hsa-miR-93-5p	27.04	0.20
hsa-miR-20b-5p	27.07	0.07
hsa-miR-150-5p	27.08	1.40
hsa-miR-193a-5p	27.13	0.01
hsa-miR-629-3p	27.14	0.69
hsa-miR-532-5p	27.16	0.38
hsa-miR-532-3p	27.26	0.38
hsa-miR-708-5p	27.28	0.27
hsa-miR-139-5p	27.28	0.98
hsa-miR-103a-3p	27.30	0.57
hsa-miR-493-3p	27.34	0.35
hsa-miR-770-5p	27.34	0.36
hsa-miR-766-3p	27.42	0.31
hsa-miR-28-5p	27.47	0.73
hsa-miR-224-5p	27.51	0.11
hsa-miR-487b-3p	27.54	0.38
hsa-miR-143-3p	27.63	0.20
hsa-miR-22-3p	27.66	1.02
hsa-let-7a-5p	27.68	0.65
hsa-miR-571	27.76	0.12
hsa-miR-15b-5p	27.83	0.76

HLSC EVs		
miRNA name	CT mean	SD
hsa-miR-93-3p	27.84	0.06
hsa-miR-495-3p	27.92	0.35
hsa-miR-30a-5p	28.01	0.85
hsa-miR-324-3p	28.05	1.04
hsa-miR-151a-5p	28.08	0.82
hsa-miR-26b-5p	28.16	0.15
hsa-miR-140-3p	28.16	0.47
hsa-miR-410-3p	28.17	0.35
hsa-miR-323a-3p	28.24	0.40
hsa-miR-628-5p	28.24	0.60
hsa-miR-425-3p	28.26	0.15
hsa-miR-452-5p	28.29	0.83
hsa-miR-1290	28.33	1.89
hsa-miR-328-3p	28.40	0.15
hsa-miR-222-5p	28.47	1.69
hsa-miR-29c-3p	28.47	1.34
hsa-miR-491-5p	28.49	0.49
hsa-miR-663b	28.61	0.19
hsa-miR-1254	28.62	0.09
hsa-miR-130b-3p	28.64	0.32
hsa-miR-1247-5p	28.69	1.75
hsa-miR-590-3p	28.76	0.22
hsa-miR-27b-3p	28.78	0.21
hsa-miR-501-5p	29.01	0.07
hsa-miR-130a-3p	29.01	0.88
hsa-miR-25-3p	29.09	0.51
hsa-miR-200c-3p	29.12	0.24
hsa-miR-1285-3p	29.13	0.37
hsa-miR-196b-5p	29.13	0.05

HLSC EVs		
miRNA name	CT mean	SD
hsa-miR-133a-3p	29.22	0.04
hsa-miR-185-5p	29.23	0.00
hsa-miR-485-5p	29.24	0.20
hsa-miR-650	29.29	1.07
hsa-miR-485-3p	29.29	0.56
hsa-miR-362-5p	29.34	0.19
hsa-miR-34b-3p	29.40	1.75
hsa-miR-27a-5p	29.40	1.29
hsa-miR-137	29.42	1.33
hsa-miR-192-5p	29.43	0.07
hsa-miR-18a-5p	29.44	0.12
hsa-miR-625-5p	29.49	1.09
hsa-miR-655-3p	29.49	0.23
hsa-miR-181a-2-3p	29.52	0.77
hsa-miR-95-3p	29.53	0.09
hsa-miR-433-3p	29.57	0.45
hsa-miR-1233-3p	29.60	3.82
hsa-miR-598-3p	29.67	0.27
hsa-miR-423-5p	29.72	0.65
hsa-miR-454-5p	29.72	0.57
hsa-miR-339-5p	29.74	0.86
hsa-miR-652-3p	29.74	0.37
hsa-miR-124-3p	29.78	1.01
hsa-miR-181c-5p	29.80	0.25
hsa-miR-335-5p	29.80	0.73
hsa-miR-337-5p	29.84	1.00
hsa-miR-455-5p	29.85	0.70
hsa-miR-136-3p	29.87	0.77
hsa-miR-296-5p	29.90	0.49

HLSC EVs		
miRNA name	CT mean	SD
hsa-miR-21-3p	29.98	1.07
hsa-miR-424-3p	30.00	0.67
hsa-miR-301a-3p	30.01	0.24
hsa-miR-324-5p	30.04	0.53
hsa-miR-22-5p	30.07	0.55
hsa-miR-214-5p	30.09	0.93
hsa-miR-148b-3p	30.13	3.57
hsa-miR-126-5p	30.16	1.52
hsa-miR-628-3p	30.20	0.04
hsa-miR-887-3p	30.21	0.23
hsa-miR-483-3p	30.24	0.56
hsa-miR-589-3p	30.28	0.42
hsa-miR-335-3p	30.28	0.10
hsa-miR-30d-5p	30.28	0.25
hsa-miR-15a-5p	30.31	0.14
hsa-miR-1271-5p	30.35	0.14
hsa-miR-125a-3p	30.37	0.10
hsa-miR-501-3p	30.40	0.70
hsa-miR-23a-3p	30.46	0.86
hsa-miR-486-5p	30.52	1.23
hsa-miR-505-3p	30.54	0.04
hsa-miR-10b-3p	30.56	0.49
hsa-miR-500a-5p	30.58	0.29
hsa-miR-330-3p	30.61	0.15
hsa-miR-636	30.75	0.27
hsa-miR-148a-3p	30.76	1.22
hsa-miR-1180-3p	30.81	0.63
hsa-miR-451a	30.86	0.99
hsa-miR-125b-1-3p	30.98	0.43

HLSC EVs		
miRNA name	CT mean	SD
hsa-miR-654-5p	31.05	0.51
hsa-miR-27b-5p	31.08	0.40
hsa-miR-1226-5p	31.08	0.11
hsa-miR-361-5p	31.18	1.73
hsa-miR-489-3p	31.26	0.61
hsa-miR-148b-5p	31.26	0.93
hsa-miR-542-5p	31.30	0.16
hsa-miR-340-5p	31.31	0.59
hsa-let-7c-5p	31.31	0.29
hsa-miR-378	31.33	0.13
hsa-miR-889-3p	31.34	0.73
hsa-miR-1227-3p	31.53	0.11
hsa-miR-505-5p	31.54	0.36
hsa-miR-320b	31.60	0.30
hsa-miR-128-3p	31.61	0.47
hsa-miR-361-3p	31.62	0.23
hsa-miR-424-5p	31.65	0.22
hsa-miR-1270	31.70	0.70
hsa-miR-1262	31.73	0.61
hsa-miR-26a-1-3p	31.73	0.64
hsa-miR-941	31.78	1.23
hsa-miR-1303	31.81	1.35
hsa-miR-409-5p	31.81	0.83
hsa-miR-503-5p	31.81	1.19
hsa-miR-543	31.84	0.60
hsa-miR-29c-5p	31.85	1.65
hsa-miR-518d-3p	31.90	0.66
hsa-miR-193a-3p	31.93	0.17
hsa-miR-18b-5p	31.95	0.28

HLSC EVs		
**miRNA name**	**CT mean**	**SD**
hsa-miR-584-5p	31.96	1.46
hsa-miR-769-5p	31.97	0.00
hsa-miR-331-5p	32.01	2.65
hsa-miR-191-3p	32.03	0.29
hsa-miR-199a-5p	32.06	2.45
hsa-miR-181a-3p	32.08	3.54
hsa-miR-130b-5p	32.08	1.24
hsa-miR-550a-3p	32.11	0.44
hsa-miR-7-2-3p	32.12	0.87
hsa-miR-885-5p	32.13	1.49
hsa-miR-198	32.18	0.65
hsa-miR-942-5p	32.20	0.35
hsa-miR-616-3p	32.24	0.49
hsa-miR-758-3p	32.28	1.40
hsa-miR-1225-3p	32.30	3.15
hsa-miR-520c-3p	32.31	0.10
hsa-miR-1291	32.31	1.51
hsa-miR-26b-3p	32.43	0.18
hsa-miR-1244	32.45	1.27
hsa-miR-671-3p	32.46	0.65
hsa-miR-642a-5p	32.46	0.63
hsa-miR-29b-3p	32.48	2.72
hsa-miR-1208	32.49	1.76
hsa-miR-203a-3p	32.49	1.19
hsa-miR-144-3p	32.49	0.23
hsa-miR-24-2-5p	32.52	0.08
hsa-miR-100-3p	32.52	2.02
hsa-miR-629-5p	32.66	1.95
hsa-miR-1201	32.68	0.07

HLSC EVs		
**miRNA name**	**CT mean**	**SD**
hsa-miR-301b-3p	32.76	1.72
hsa-miR-411-3p	32.79	2.37
hsa-miR-135b-5p	32.85	0.45
hsa-miR-744-3p	32.85	0.16
hsa-miR-194-5p	32.85	0.50
hsa-miR-422a	32.91	1.52
hsa-miR-380-5p	32.93	3.67
hsa-miR-601	33.11	0.74
hsa-miR-638	33.12	3.32
hsa-miR-497-5p	33.13	0.53
hsa-miR-449b-5p	33.15	0.86
hsa-miR-576-3p	33.15	0.59
hsa-miR-604	33.16	2.08
hsa-miR-23b-3p	33.21	1.18
hsa-miR-449a	33.22	1.71
hsa-miR-19b-1-5p	33.23	0.97
hsa-miR-455-3p	33.45	0.56
hsa-miR-622	33.47	0.47
hsa-miR-502-3p	33.51	1.09
hsa-miR-18a-3p	33.53	0.30
hsa-miR-938	33.55	0.53
hsa-miR-369-5p	33.62	0.57
hsa-miR-616-5p	33.62	0.74
hsa-miR-1825	33.63	0.77
hsa-miR-10b-5p	33.65	1.95
hsa-miR-106b-3p	33.67	0.30
hsa-miR-1255b-5p	33.69	0.81
hsa-miR-193b-5p	33.72	0.62
hsa-miR-450b-5p	33.77	0.54

HLSC EVs		
**miRNA name**	**CT mean**	**SD**
hsa-miR-668-3p	33.78	1.42
hsa-miR-9-5p	33.85	0.12
hsa-miR-520b	34.00	0.27
hsa-miR-187-3p	34.03	1.03
hsa-miR-101-3p	34.12	1.22
hsa-miR-10a-3p	34.14	1.12
hsa-miR-502-5p	34.15	0.35
hsa-miR-603	34.20	0.09
hsa-miR-517c-3p	34.29	0.83
hsa-miR-184	34.31	0.38
hsa-miR-340-3p	34.31	1.05
hsa-miR-16-1-3p	34.36	0.70
hsa-miR-656-3p	34.39	1.17
hsa-miR-33a-5p	34.42	7.89
hsa-miR-1305	34.45	0.79

Supplementary Table S3. List of miRNAs carried by HLSC EVs. Data are displayed as CT mean value ± SD (n=3). The 15 most expressed miRNAs, used in bioinformatics analysis, are highlighted in bold.

Supplementary Table S4. List of miRNAs vehicle by FIBRO EVs.

FIBRO EVs		
**miRNA name**	**CT mean**	**SD**
**hsa-miR-24-3p**	23.12	0.24
**hsa-miR-484**	24.18	0.30
**hsa-miR-222-3p**	24.33	0.24
**hsa-miR-1233-3p**	24.62	0.29
**hsa-miR-574-3p**	24.68	0.64
**hsa-miR-409-3p**	24.84	1.06
**hsa-miR-1247-5p**	24.93	0.71
**hsa-miR-191-5p**	24.97	0.43
**hsa-miR-320a**	25.23	0.02
**hsa-miR-29a-3p**	25.37	0.05
**hsa-miR-193b-3p**	25.74	0.75
**hsa-miR-31-5p**	26.17	0.57
**hsa-miR-19b-3p**	26.19	0.03
**hsa-miR-376a-3p**	26.25	0.95
**hsa-miR-16-5p**	26.27	0.90
hsa-miR-596	26.27	0.45
hsa-miR-376c-3p	26.31	0.25
hsa-miR-1260a	26.51	1.79
hsa-miR-1225-3p	26.51	1.17
hsa-miR-197-3p	26.54	0.30
hsa-miR-214-3p	26.78	0.87
hsa-miR-149-5p	26.96	1.39
hsa-miR-155-5p	26.99	0.40
hsa-miR-571	27.03	0.08
hsa-miR-17-5p	27.04	0.06
hsa-miR-1825	27.15	0.44
hsa-miR-106a-5p	27.31	0.01
hsa-miR-370-3p	27.33	0.72
hsa-let-7b-5p	27.34	0.35

FIBRO EVs		
**miRNA name**	**CT mean**	**SD**
hsa-let-7e-5p	27.36	0.14
hsa-miR-99a-5p	27.39	0.90
hsa-miR-199a-3p hsa-miR-199b-3p	27.42	1.18
hsa-miR-146a-5p	27.51	0.06
hsa-miR-1267	27.54	6.38
hsa-miR-432-5p	27.60	0.28
hsa-miR-663b	27.72	0.81
hsa-let-7b-3p	27.75	1.07
hsa-miR-134-5p	27.95	0.15
hsa-miR-454-3p	27.97	0.56
hsa-miR-100-5p	28.00	0.10
hsa-miR-331-3p	28.04	0.41
hsa-miR-186-5p	28.05	0.01
hsa-miR-374b-5p	28.26	0.06
hsa-miR-30a-3p	28.30	0.60
hsa-miR-21-5p	28.41	0.01
hsa-miR-342-3p	28.41	0.62
hsa-miR-345-5p	28.52	0.19
hsa-miR-30e-3p	28.56	0.28
hsa-miR-138-5p	28.64	0.39
hsa-miR-1227-3p	28.64	0.58
hsa-miR-30c-5p	28.79	0.07
hsa-miR-411-5p	28.84	0.20
hsa-miR-221-3p	28.87	0.60
hsa-miR-145-5p	28.98	0.17
hsa-miR-125a-5p	28.98	0.18
hsa-miR-28-3p	28.99	0.25
hsa-miR-146b-5p	29.03	0.94
hsa-miR-34b-3p	29.07	0.84

FIBRO EVs		
**miRNA name**	**CT mean**	**SD**
hsa-miR-222-5p	29.08	0.86
hsa-miR-770-5p	29.11	0.98
hsa-miR-20a-5p	29.12	0.42
hsa-miR-26a-5p	29.13	0.21
hsa-miR-30b-5p	29.17	0.26
hsa-miR-151a-3p	29.33	0.18
hsa-miR-328-3p	29.34	1.83
hsa-miR-133a-3p	29.35	0.22
hsa-miR-661	29.37	0.27
hsa-miR-625-3p	29.41	0.46
hsa-miR-539-5p	29.42	0.76
hsa-miR-34a-5p	29.45	0.64
hsa-miR-374a-5p	29.46	0.03
hsa-miR-382-5p	29.53	0.79
hsa-miR-425-5p	29.63	1.08
hsa-miR-125b-5p	29.65	0.02
hsa-miR-1290	29.65	0.48
hsa-miR-495-3p	29.67	0.02
hsa-miR-744-5p	29.71	0.25
hsa-let-7g-5p	29.71	0.00
hsa-miR-126-5p	29.76	0.01
hsa-miR-31-3p	29.82	0.53
hsa-miR-605-5p	29.96	2.13
hsa-miR-92a-3p	30.00	0.08
hsa-miR-30a-5p	30.08	0.50
hsa-miR-99b-5p	30.17	0.35
hsa-miR-1270	30.28	1.06
hsa-miR-1303	30.31	0.38
hsa-miR-212-3p	30.39	1.78

FIBRO EVs		
**miRNA name**	**CT mean**	**SD**
hsa-miR-494-3p	30.39	0.02
hsa-miR-99b-3p	30.42	0.48
hsa-miR-106b-5p	30.44	0.35
hsa-miR-1254	30.47	0.50
hsa-let-7d-5p	30.49	0.25
hsa-miR-339-3p	30.52	0.66
hsa-miR-668-3p	30.62	0.44
hsa-miR-7-1-3p	30.65	2.57
hsa-miR-433-3p	30.69	0.43
hsa-miR-143-3p	30.71	0.95
hsa-miR-181a-5p	30.74	0.36
hsa-miR-93-5p	30.78	1.05
hsa-miR-19a-3p	30.81	0.35
hsa-miR-638	30.82	0.61
hsa-miR-659-3p	30.83	0.51
hsa-miR-335-3p	30.84	2.04
hsa-miR-493-3p	30.85	0.25
hsa-miR-1271-5p	30.88	1.55
hsa-miR-27a-3p	30.91	0.89
hsa-miR-410-3p	30.93	0.41
hsa-miR-152-3p	30.93	1.01
hsa-miR-34a-3p	30.97	0.90
hsa-miR-425-3p	31.01	0.28
hsa-miR-636	31.03	0.06
hsa-miR-10a-5p	31.04	0.08
hsa-miR-26b-5p	31.17	1.11
hsa-miR-150-5p	31.17	0.82
hsa-miR-223-3p	31.18	0.55
hsa-miR-324-3p	31.22	0.20

FIBRO EVs		
**miRNA name**	**CT mean**	**SD**
hsa-miR-140-5p	31.26	1.27
hsa-miR-487b-3p	31.35	1.00
hsa-miR-136-3p	31.35	2.64
hsa-miR-655-3p	31.47	0.41
hsa-miR-1208	31.51	1.17
hsa-miR-483-5p	31.62	0.52
hsa-miR-379-5p	31.65	0.59
hsa-miR-195-5p	31.66	0.64
hsa-miR-943	31.67	0.23
hsa-miR-103a-3p	31.72	1.40
hsa-miR-590-5p	31.82	0.08
hsa-miR-335-5p	31.98	0.51
hsa-miR-132-3p	31.99	0.42
hsa-miR-708-5p	32.04	0.02
hsa-miR-144-3p	32.09	2.51
hsa-miR-15b-5p	32.12	1.04
hsa-miR-20b-5p	32.17	0.61
hsa-miR-323a-3p	32.20	0.10
hsa-miR-378a-3p	32.24	0.35
hsa-miR-93-3p	32.24	0.39
hsa-miR-1275	32.29	1.06
hsa-miR-28-5p	32.49	1.10
hsa-miR-629-3p	32.57	0.17
hsa-miR-485-3p	32.57	0.49
hsa-miR-218-5p	32.61	0.85
hsa-miR-1226-5p	32.67	2.87
hsa-miR-431-5p	32.76	1.19
hsa-miR-516b-3p	32.79	0.85
hsa-miR-365a-3p hsa-miR-365b-3p	32.84	0.21

FIBRO EVs		
**miRNA name**	**CT mean**	**SD**
hsa-miR-532-3p	32.97	0.61
hsa-miR-628-5p	33.01	1.37
hsa-miR-892b	33.02	2.16
hsa-miR-1291	33.02	0.29
hsa-miR-520b	33.05	0.71
hsa-miR-151a-5p	33.08	2.04
hsa-miR-29b-3p	33.11	0.34
hsa-miR-196b-5p	33.16	0.82
hsa-miR-130b-3p	33.22	4.69
hsa-miR-769-5p	33.24	5.66
hsa-miR-564	33.29	0.06
hsa-miR-424-3p	33.30	0.96
hsa-miR-455-5p	33.32	1.31
hsa-miR-889-3p	33.33	0.71
hsa-miR-200c-3p	33.44	5.56
hsa-miR-543	33.44	0.70
hsa-miR-650	33.44	6.21
hsa-miR-337-5p	33.44	0.19
hsa-miR-491-5p	33.45	6.13
hsa-miR-27a-5p	33.47	5.16
hsa-miR-25-3p	33.50	6.48
hsa-miR-338-5p	33.55	0.71
hsa-miR-214-5p	33.55	5.18
hsa-miR-320b	33.55	6.08
hsa-miR-140-3p	33.66	5.33
hsa-miR-340-5p	33.76	1.61
hsa-miR-26a-1-3p	33.82	1.24
hsa-miR-598-3p	33.90	1.28
hsa-miR-324-5p	33.91	5.97

FIBRO EVs		
**miRNA name**	**CT mean**	**SD**
hsa-miR-33a-5p	33.93	6.77
hsa-miR-589-3p	33.94	5.29
hsa-miR-520c-3p	33.98	0.62
hsa-miR-601	34.00	0.50
hsa-miR-29c-3p	34.16	0.26
hsa-miR-548c-3p	34.16	0.35
hsa-miR-18a-5p	34.17	0.26
hsa-miR-193a-5p	34.24	0.39
hsa-miR-301a-3p	34.33	4.99
hsa-miR-130a-3p	34.38	0.73
hsa-miR-939-5p	34.38	7.95
hsa-miR-486-5p	34.42	0.31
hsa-miR-125a-3p	34.48	0.24
hsa-miR-148b-3p	34.54	2.67

Supplementary Table S4. List of miRNAs carried by FIBRO EVs. Data are displayed as CT mean value ± SD (n=3). The 15 most expressed miRNAs, used in bioinformatics analysis, are highlighted in bold.

Supplementary Table S5. List of primers used for qRT-PCR experiments.

Gene symbol	Forward	Reverse
m_Col1	ATCTCCTGGTGCTGATGGAC	ACCTTGTTTGCCAGGTTCAC
m_Tgfb1	CGAAAGCCCTGTATTCCGTCT	GCAACAATTCCTGGCGTTACC
m_α-Sma	CTGACAGAGGCACCACTGAA	CATCTCCAGAGTCCAGCACA
m_Gapdh	TGTCAAGCTCATTTCTGGTATGA	TCTTACTCCTTGGAGGCCATGT

Supplementary Table S5. List of primers used to evaluate pro-fibrotic gene expressions in healthy mice and in DN mice treated or not with EVs by qRT-PCR.
